# Anticancer Effects of Abietane Diterpene 7α-Acetoxy-6β-hydroxyroyleanone from *Plectranthus grandidentatus* and Its Semi-Synthetic Analogs: An In Silico Computational Approach

**DOI:** 10.3390/molecules29081807

**Published:** 2024-04-16

**Authors:** Vera M. S. Isca, Przemysław Sitarek, Anna Merecz-Sadowska, Magdalena Małecka, Monika Owczarek, Joanna Wieczfińska, Radosław Zajdel, Paweł Nowak, Patricia Rijo, Tomasz Kowalczyk

**Affiliations:** 1 Center for Research in Biosciences & Health Technologies (CBIOS), Universidade Lusófona de Humanidades e Tecnologias, 1749-024 Lisbon, Portugal; vera.isca@ulusofona.pt; 2 Department of Medical Biology, Medical University of Lodz, Muszynskiego 1, 90-151 Lodz, Poland; przemyslaw.sitarek@umed.lodz.pl; 3 Department of Economic and Medical Informatics, University of Lodz, 90-214 Lodz, Poland; anna.merecz-sadowska@uni.lodz.pl (A.M.-S.); radoslaw.zajdel@uni.lodz.pl (R.Z.); pawel.nowak@uni.lodz.pl (P.N.); 4 Department of Allergology and Respiratory Rehabilitation, Medical University of Lodz, 90-725 Lodz, Poland; 5 Department of Physical Chemistry, Faculty of Chemistry, University of Lodz, Pomorska 163/165, 90-236 Lodz, Poland; magdalena.malecka@chemia.uni.lodz.pl; 6 Łukasiewicz Research Network, Lodz Institute of Technology, Skłodowskiej-Curie 19/27, 90-570 Lodz, Poland; monika.owczarek@lit.lukasiewicz.gov.pl; 7 Department of Immunopathology, Medical University of Lodz, Zeligowskiego 7/9, 90-752 Lodz, Poland; joanna.wieczfinska@umed.lodz.pl; 8 Department of Medical Informatics and Statistics, Medical University of Lodz, 90-645 Lodz, Poland; 9 Instituto de Investigação do Medicamento (iMed.ULisboa), Faculdade de Farmácia, Universidade de Lisboa, 1649-003 Lisbon, Portugal; 10 Department of Molecular Biotechnology and Genetics, Faculty of Biology and Environmental Protection, University of Lodz, Banacha 12/16, 90-237 Lodz, Poland

**Keywords:** 7α-acetoxy-6β-hydroxyroyleanone, *Plectranthus grandidentatus*, anticancer activity

## Abstract

The abietane diterpenoid 7α-acetoxy-6β-hydroxyroyleanone (Roy) isolated from *Plectranthus grandidentatus* demonstrates cytotoxicity across numerous cancer cell lines. To potentiate anticancer attributes, a series of semi-synthetic Roy derivatives were generated and examined computationally. ADMET predictions were used to evaluate drug-likeness and toxicity risks. The antineoplastic potential was quantified by PASS. The DFT models were used to assess their reactivity and stability. Molecular docking determined cancer-related protein binding. MS simulations examined ligand–protein stability. Additionally, network pharmacology was used to identify potential targets and signaling pathways. Favorable ADME attributes and acceptable toxicity profiles were determined for all compounds. Strong anticancer potential was shown across derivatives (Pa 0.819–0.879). Strategic modifications altered HOMO–LUMO gaps (3.39–3.79 eV) and global reactivity indices. Favorable binding was revealed against cyclin-dependent kinases, BCL-2, caspases, receptor tyrosine kinases, and p53. The ligand exhibited a stable binding pose in MD simulations. Network analysis revealed involvement in cancer-related pathways. In silico evaluations predicted Roy and derivatives as effective molecules with anticancer properties. Experimental progress is warranted to realize their chemotherapeutic potential.

## 1. Introduction

Medicinal plants are an invaluable source of bioactive compounds with a wide range of therapeutic properties [1,2]. While cancer is one of the leading causes of death worldwide and mortality rates continue to rise, many malignancies lack effective treatments. Chemotherapy can fail due to intrinsic or acquired drug resistance coupled with prohibitive side effects. Therefore, there is an unmet need for novel therapeutic agents to overcome these limitations [3,4,5]. Natural products possess an unparalleled structural diversity and potential as modulators of biomolecular function that may serve as an essential source for drug discovery [6].

Lamiaceae, one of the most ubiquitous and ecologically diverse plant families, represent an abundant reservoir of bioactive specialized metabolites [7,8]. The genus *Plectranthus* (Lamiaceae) encompasses around eighty species worldwide, many of which are prominently mentioned in traditional medicine [9]. Numerous in vitro and in vivo studies have shown that both extracts and pure compounds isolated from the genus *Plectranthus* exhibit a range of biological properties such as anticancer, antioxidant, anti-inflammatory, antimicrobial, etc. [10,11]. Acetone extract from *P. madagascariensis* revealed significant antibacterial activity against Gram-positive bacteria (MIC: 1.95 < MIC < 7.81 μg/mL), inclusive of a strain of methicillin-resistant *Staphylococcus aureus* (MRSA). Moreover, acetone extracts from both *P. madagascariensis* and *P. neochilus* demonstrated noteworthy antibacterial efficacy against Gram-negative *Klebsiella pneumonia* (0.48 < MIC < 3.91 μg/mL), confirming the traditional utilization of these plants as anti-infectious agents. All methanolic extracts exhibited potent antioxidant properties at a concentration of 100 μg/mL, as determined by their radical scavenging activity (60.8–89.0%) in the 2,2-diphenyl-1-picrylhydrazyl (DPPH) assay. The acetone extract from *P. madagascariensis* obtained via maceration exhibited moderate cytotoxic effects in the MDA-MB-231 cell line (triple-negative human breast carcinoma) [12].

Although the phytochemical diversity of the genus has not been fully explored yet, diterpenoids constitute the predominant bioactive components, with over 140 highly functionalized abietane derivatives characterized thus far [13,14]. The anticancer properties of the *Plectranthus* abietane diterpenes have garnered particular attention, with prospective modes of action centered around the induction of apoptosis triggered by intrinsic and extrinsic mitochondrial pathways [15,16]. As part of our ongoing investigations of the anticancer potential of secondary metabolites of *Plectranthus*, we have recently isolated 7α-acetoxy-6β-hydroxyroyleanone (Roy) from *P. madagascariensis*, which showed intriguing bioactivity on a range of assay platforms. In an effort to potentiate and refine the preliminary pharmacology surrounding Roy, we embarked on a semi-synthesis initiative to access structurally related analogs for comparative screening. Herein, we evaluate the anticancer effects for lead candidate Roy’s semi-synthetic analogs using computational simulations, providing key insights to guide and accelerate subsequent phases of experimental validation.

This study aimed to comprehensively evaluate the anticancer potential of five semi-synthetic analogs of the abietane diterpenoid 7α-acetoxy-6β-hydroxyroyleanone using in silico methodologies. We assessed pharmacokinetic profiles, drug-like attributes, and toxicity risks. The derivatives were subjected to density functional theory computations to determine equilibrium geometries and electronic features. Molecular docking provided insight into ligand binding against protein targets implicated in carcinogenesis. Subsequently, molecular dynamics simulations examined the temporal stability of ligand–protein complexes. Additionally, network pharmacology approaches were utilized to construct protein–protein interaction networks and identify signaling pathways. Utilizing computational methods will enable the demonstration of the most favorable in silico semi-synthetic abietane analogs’ anticancer potential for targeted validation through in vitro experimentation.

## 2. Results

### 2.1. Compounds

**Figure 1:**
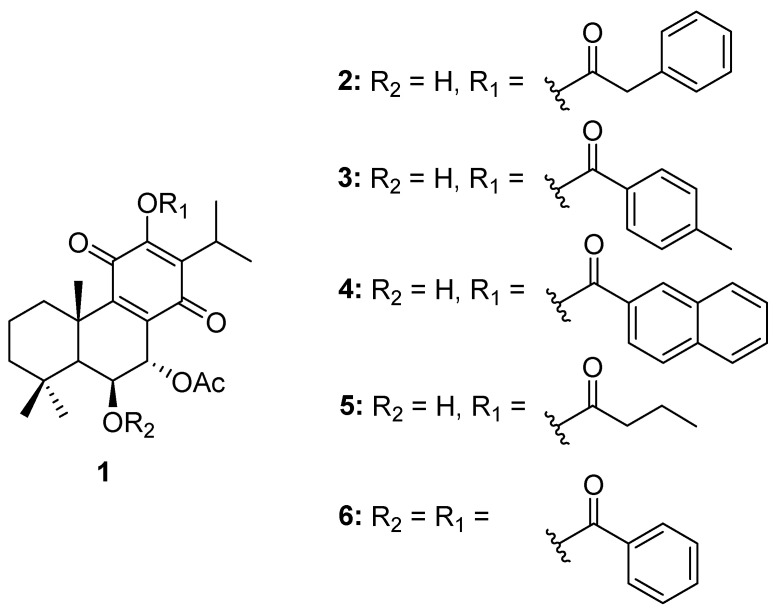
Compound **1** and derivatives (**2**–**6**) synthesized from **1** (**Roy**).

The chemical structures of compound **1** (**Roy**) and its semi-synthetic derivatives (**2**–**6**) are presented in Figure 1.

### 2.2. ADMET and Drug-Likeness Analysis Results

The physicochemical parameters of the examined compounds predicted through the SwissADME platform are presented in Table S1. Molecular weight (MW) values ranged from 390.47 to 598.68 g/mol. Compounds **2**, **3**, and **6** have a molecular weight greater than 500 g/mol.

The lipophilicity parameters of the examined compounds predicted through the SwissADME platform are presented in Table S2. The computed consensus log P values (lipophilicity) for the selected compounds ranged from 2.83 to 5.86. The lipophilicity of most compounds is below **5**, except compound **6**. This indicates that these molecules could be explored as orally active agents, emphasizing the critical importance of this physicochemical property in pharmacokinetics and drug discovery.

The water-solubility parameters of the examined compounds predicted through the SwissADME platform are presented in Table S3. The computed water solubility index indicates that screened compounds exhibit a range of water solubility, from moderately soluble to poorly soluble, indicating that efforts should be made to enhance solubility during the formulation process.

The pharmacokinetic parameters of the examined compounds predicted through the SwissADME platform are presented in Table S4. High gastrointestinal absorption was observed for compounds **1**–**5**. Blood–brain barrier (BBB) permeability potential was not predicted for all compounds. BBB permeability potential is a parameter that predicts a compound’s ability to cross the blood–brain barrier, which is crucial for drugs targeting the central nervous system. All the compounds showed the potential to be substrates for P-gp. Inhibitory potential against cytochrome P450 (CYP) isoforms was observed for compounds **2**, **3**, and **6** (for two isoforms) and for compounds **1**, **4**, and **5** (for one isoform).

The drug-likeness, medicinal chemistry, and lead-likeness parameters of the examined compounds predicted through the SwissADME platform are presented in Table S5. Compounds **1** and **5** showed no violation of all five filters (Lipinski, Ghose, Veber, Egan, and Muegge) used. The other compounds exhibited violations of at least one of the drug-likeness filters. The calculated bioavailability score for all compounds placed them within the 56% probability class. All compounds showed at least one PAINS or BRENK alert and a violation of lead-likeness.

### 2.3. Toxicity Prediction Results

The lethal dose, expressed in milligrams per kilogram, representing the amount of the compounds estimated to be fatal to 50% of rats in acute oral toxicity studies, as well as the predicted toxicity categories ranging from class I to VI and the associated prediction accuracy as a percentage obtained utilizing the ProTox-II server for the screened chemicals, is provided in Table 1. Compounds **2** and **5** exhibited the highest toxicity (LD50 = 75 mg/kg), classified as class III (50 < LD50 ≤ 300), indicating potential toxicity risks upon oral exposure. Compound **1** demonstrated the lowest toxicity (LD50 = 1000 mg/kg), categorized as class IV (300 < LD50 ≤ 2000), representing compounds most likely to cause harm when orally exposed. None of the analyzed compounds were classified as class 1 (LD50 ≤ 5) or class 2 (5 < LD50 ≤ 50), which correspond to agents with potential lethality. Furthermore, none of the chemicals under investigation fell into category 5 (2000 < LD50 ≤ 5000) or category 6 (LD50 > 5000). Class 5 represents compounds unlikely to induce harm upon oral exposure, while class 6 denotes nontoxic substances.

The toxicity information generated by the ProTox-II platform for the investigated molecules is provided in Table S6. None of the molecules exhibited hepatotoxicity. All agents showed carcinogenic potential, with probability values ranging from 0.50 to 0.53. Moreover, the entirety of the compounds elicited immunotoxic effects, evidenced by probability scores between 0.87 and 0.98. However, the absence of mutagenic activity was noted in the series of compounds. Likewise, cytotoxicity was not observed for the screened library.

Table S7 provides the Tox21-nuclear receptor signaling pathway parameters, including AhR, AR, AR-LBD, Aro, ER, ER-LBD, and PPAR-Gamma predictions generated through the ProTox II server for the investigated molecules. All compounds were obtained as inactive for all parameters.

Table S8 demonstrates the Tox21-stress response pathway parameters, including nrf2/ARE, HSE, MMP, p53, and ATAD5 predictions generated through the ProTox II server for the investigated molecules. Compounds **1**–**3** were obtained as inactive for all parameters. Compounds **4**–**6** were MMP-active with a probability score of 52%, 50%, and 53%, respectively.

Table S9 shows the predicted acute toxicity, including inhalation, oral, dermal, eye irritation and corrosion, skin sensitization, and skin irritation and corrosion predictions generated through the StopTox server for the investigated molecules. The compounds did not exhibit any form of acute toxicity upon exposure.

The predictions of mutagenicity, tumorigenicity, irritancy, and reproductive toxicity generated by the OSIRIS platform for the molecules studied are presented in Table S10. Moderate mutagenic risk was observed for all compounds. Moreover, the constituents were determined to elicit low tumorigenic hazard, high irritant potential, and low reproductive effectivity.

### 2.4. Antineoplastic and Anticarcinogenic Activity Results

The anticancer activity profiles for the investigated molecules as predicted by the PASS platform are shown in Table 2. Antineoplastic potential, with Pa values ranging from 0.879 to 0.819, was determined for all compounds. Furthermore, minimal predicted anticarcinogenic effects (Pa < 0.5) were concurrently calculated for the dataset. However, a comparative analysis of the Pa and Pi output revealed a Pa above 0.3 for all derivatives. Compounds were classified as potentially bioactive if they exhibited a Pa over 0.3 on the basis of precedents.

### 2.5. DFT Calculations Results

**Figure 2:**
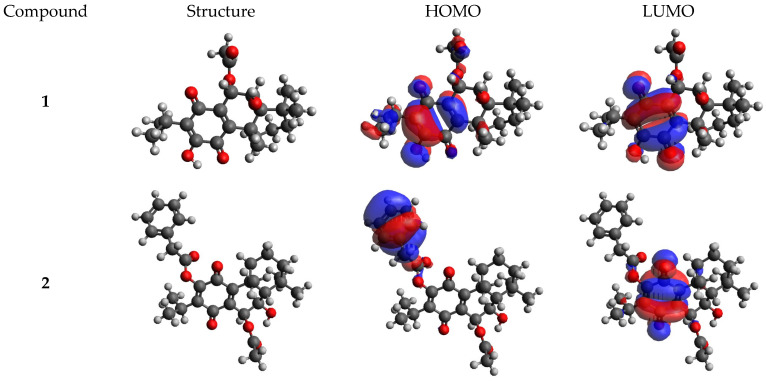

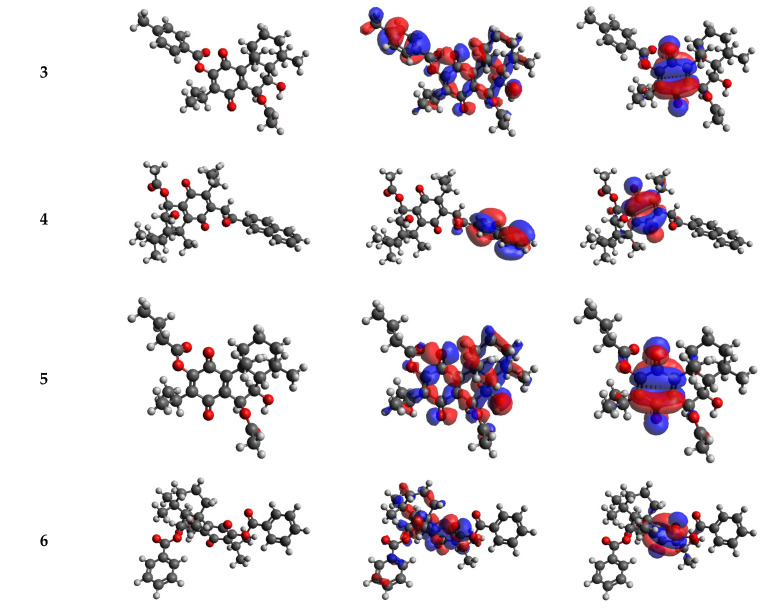
Frontier molecular orbitals (HOMO and LUMO) calculated for compounds **1**–**6**.

The highest occupied and lowest unoccupied molecular orbitals (HOMO and LUMO) provided insight into the reactivity and stability of molecules. Geometrical configurations optimized with DFT generated for compounds **1**–**6** examined are shown in Figure 2.

The HOMO and LUMO energies and global reactivity descriptors are shown in Table 3. The LUMO energy determines the electron-accepting aptitude of the molecule, whereas the HOMO energy controls the electron-donating ability [17,18,19]. Furthermore, quantum chemical parameters such as hardness (η), softness (S), electronegativity (χ), and electrophilicity (ω) are global descriptors of the chemical behavior of the molecules. The hardness value determines how the atom resists the charge transfer to another atom, while the ability of an atom to receive electrons is measured by the softness value. Electronegativity is a chemical property that describes a molecule’s tendency to attract electrons. The scale of the electrophilic property of a molecule is determined by the electrophilicity index [20,21,22,23]. Additionally, the ΔN value is an important index widely recognized to help predict molecules’ chemical reactivity and kinetic stability. A lower HOMO–LUMO gap value indicates that the molecule is more susceptible to polarization and is considered a soft molecule. Conversely, a larger energy gap characterizes a hard molecule, which is more resistant to polarization due to the higher energy required for excitation. Consequently, soft molecules with lower energy gaps exhibit higher reactivity compared to their hard counterparts as they can readily donate electrons to an acceptor [24,25].

The HOMO–LUMO gap for compounds **1**–**6** was determined to be 3.39 to 3.79 eV. The obtained band gap shown by compound **2** was lower than the band gaps of others, making it the highest chemical reactivity. A smaller band gap of a compound predicts the need for a small quantity of energy to become excited from ground level. In the case of compound **6**, the band gap was larger than others, making it the lowest chemical reactivity. Compound **2** was found to have a lower hardness and a higher softness value. Furthermore, compound **5** had the highest electronegativity index, and compound **4** had the lowest electrophilicity index value.

The chemical hardness value of the compound suggests that it may interact more readily with the active site, emphasizing the significance of ligand hydrophobicity in determining affinity with the active site, a crucial aspect to consider in the context of molecular docking studies [26,27].

### 2.6. Molecular Docking Results

**Figure 3:**
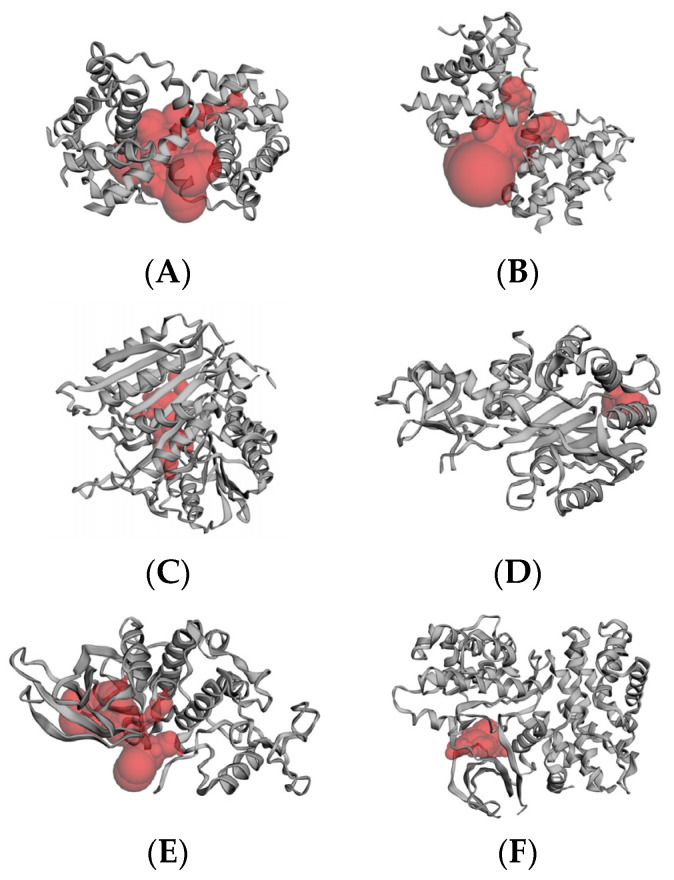

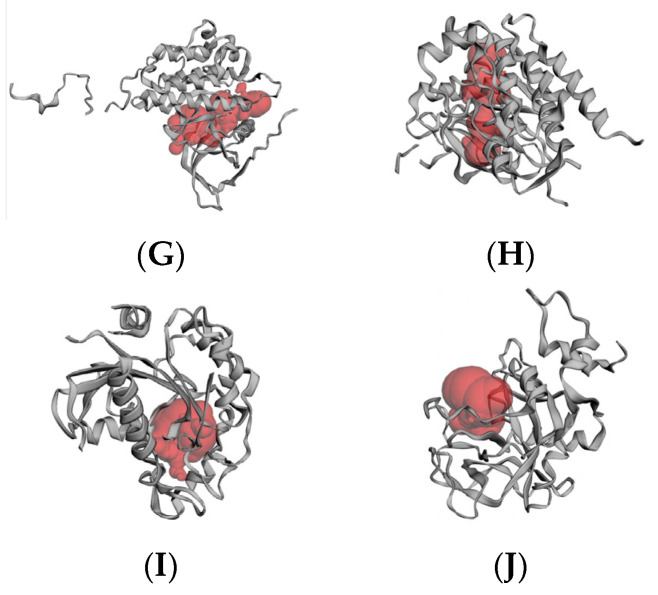
CAST-p pocket estimation: (**A**) BCL-2; (**B**) BCL-XL; (**C**) caspase 3; (**D**) caspase 9; (**E**) CDK2; (**F**) CDK6; (**G**) EGFR; (**H**) VEGFR; (**I**) p53; and (**J**) PARP-1.

The protein structures were analyzed by CAST-p, followed by the implementation of the molecular docking procedure. Ideal pocket areas (SA) for the targets BCL-2, BCL-XL, caspase 3, caspase 9, CDK2, CDK6, EGFR, VEGFR, p53, and PARP-1 were estimated to be 1097.807 Å, 1253.272 Å, 438.406 Å, 148.835 Å, 580.293 Å, 391.147 Å, 703.171 Å, 437.826 Å, 380.470 Å, and 317.230 Å, respectively. Figure 3 shows the predicted active site region of target proteins.

Molecular docking studies were conducted to examine the binding interactions between six selected compounds (**1**–**6**) and the active sites of 10 target proteins (BCL-2, BCL-XL, caspase 3, caspase 9, CDK2, CDK6, EGFR, VEGFR, p53, and PARP-1) implicated in the cancer-related pathways.

The proteins investigated in the molecular docking analyses comprised essential regulators of apoptosis, cell cycle, and growth factor signaling linked to cancer pathogenesis. These proteins included anti-apoptotic proteins BCL-2 and BCL-XL, apoptotic effector caspases 3 and 9, cell cycle-promoting CDKs 2 and 6, growth factor receptors EGFR and VEGFR, tumor suppressor p53, and DNA repair protein PARP-1. This selection of 10 cancer-related proteins represents pivotal nodes in signaling cascades regulating cell proliferation, survival, angiogenesis, and genetic stability in malignant cells. Employing structure-based investigations to target these proteins could aid in the development of selective small-molecule experimental compounds with potential anticancer effects against high-priority targets.

The docking outcomes provided the expected binding energies between ligands and proteins, measured in kcal/mol units for specific compounds associated with distinct target proteins. Specifically, compound **1** showed a strong predicted affinity for target proteins caspase 9 (complex 1) with a binding energy value of −10.60. Compound **2** for target protein BCL-2 (complex 2) had a binding energy value of −10.46 kcal/mol. Meanwhile, compound **4** exhibited binding energy values of −11.65, −12.84 kcal/mol, −11.86, −13.49 kcal/mol, 12.39 kcal/mol, and −11.40 kcal/mol for target proteins EGFR (complex 3), BCL-XL (complex 4), caspase 3 (complex 5), CDK2 (complex 6), VEGFR (complex 7), and p53 (complex 8), respectively; compound **6** exhibited a binding energy value of −15.45 kcal/mol for target proteins CDK6 (complex 9) and PARP-1 (complex 10). These are complexes of a given protein with the ligand with the highest binding energy value.

To validate the docking method, known inhibitors were also docked to the proteins BCL-2, BCL-XL, caspase 3, CDK2, CDK6, EGFR, VEGFR, and PARP-1. The inhibitors used were BCL-2 inhibitor 1-[2-[(3S)-3-(aminomethyl)-3,4-dihydro-1H-isoquinoline-2-carbonyl]phenyl]-4-chloro-5-methyl-N,N-diphenylpyrazole-3-carboxamide (−9.11 kcal/mol), BCL-XL inhibitor N-[3-[5-[(E)-N-(1,3-benzothiazol-2-ylamino)-C-methylcarbonimidoyl]furan-2-yl]phenyl]sulfonyl-6-phenylhexanamide (−14.04 kcal/mol), caspase 3 inhibitor 5-[[4-[[(2S)-1-carboxy-3-oxobutan-2-yl]carbamoyl]phenyl]methylsulfamoyl]-2-hydroxybenzoic acid (−10.58 kcal/mol), CDK2 inhibitor 3-[(6,7-dimethoxyquinazolin-4-yl)amino]phenol (−9.72 kcal/mol), CDK6 inhibitor 2-(3,4-dihydroxyphenyl)-3,7-dihydroxychromen-4-one (−10.86 kcal/mol), EGFR inhibitor N-(3-ethynylphenyl)-6,7-bis(2-methoxyethoxy)quinazolin-4-aminE (−8.87 kcal/mol), VEGFR inhibitor methyl N-[6-[4-[[2-fluoro-5-(trifluoromethyl)phenyl]carbamoylamino]phenoxy]-1H-benzimidazol-2-yl]carbamate (−15.33 kcal/mol), and PARP-1 inhibitor 4-[[3-[4-(cyclopropanecarbonyl)piperazine-1-carbonyl]-4-fluorophenyl]methyl]-2H-phthalazin-1-one (−13.71 kcal/mol). The structures of these inhibitors were obtained from the Protein Data Bank (PDB), where they were co-crystallized with their respective target proteins. The binding energies of these known inhibitors provide a reference point for assessing the predicted affinities of the studied compounds **1**–**6**.

**Figure 4:**
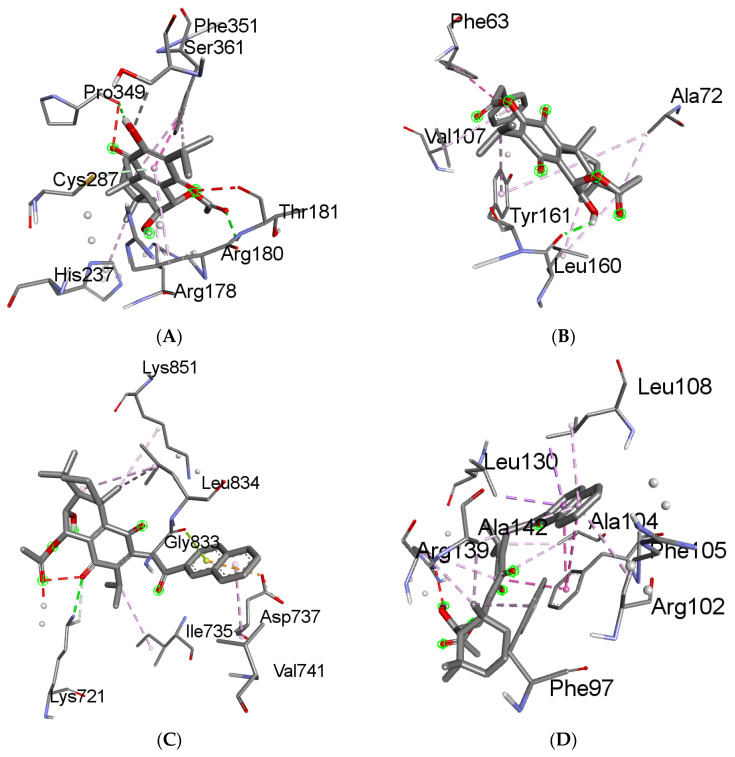

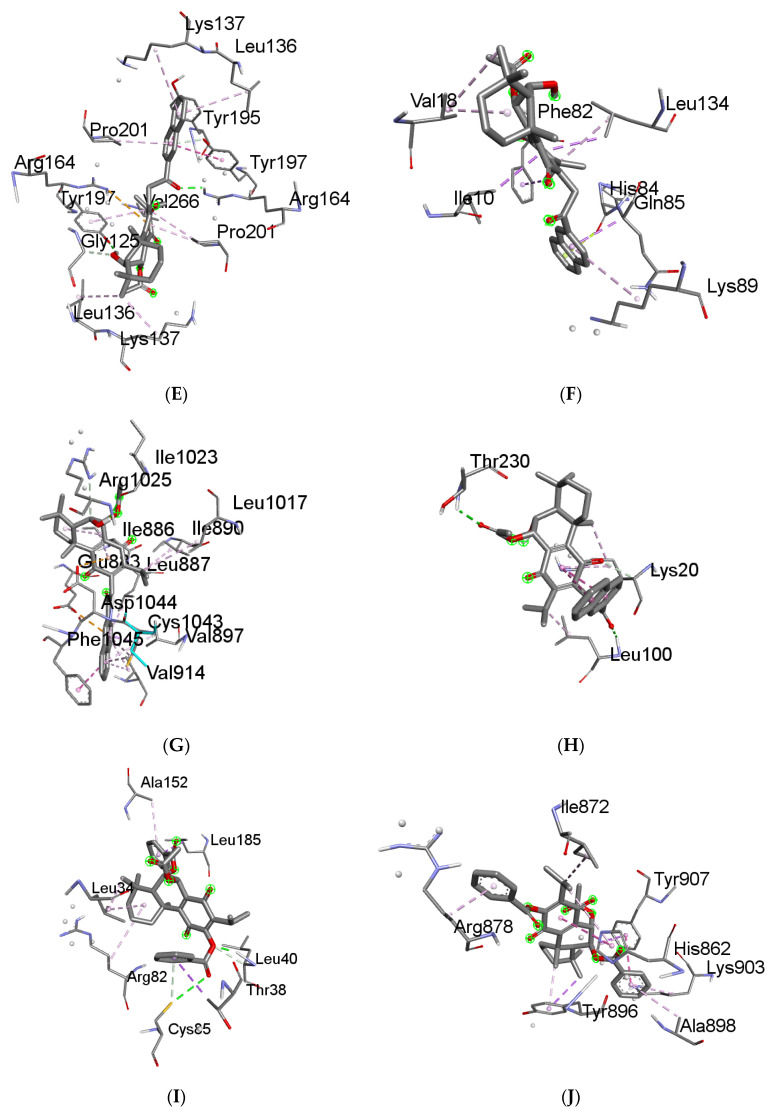
The 3D interactions of compound **1** against caspase 9—complex 1 (**A**); compound **2** against BCL-2—complex 2 (**B**); compound **4** against EGFR—complex 3 (**C**), BCL-XL—complex 4 (**D**), caspase 3—complex 5 (**E**), CDK2—complex 6 (**F**), VEGFR—complex 7 (**G**), p53—complex 8 (**H**); compound **6** against CDK6—complex 9 (**I**); and PARP-1—complex 10 (**J**).

The degree of negativity in the binding energy values reflects the favorable binding potential between the compounds and the target proteins. Table S11 provides an in-depth examination of the docking poses, highlighting the crucial molecular interactions that may contribute to the strong predicted affinities of compounds for their respective targets. The binding modes and interactions between ligands and proteins (complex 1–10) are visually represented in Figure 4.

### 2.7. Molecular Dynamics Simulation Results

The dynamic aspects of the docking complexes 1–10 were simulated for 100 ns. MDSs were applied to investigate the stability of the proteins and ligands during their interaction. This was performed by calculating and analyzing the RMSD parameter.

**Figure 5:**
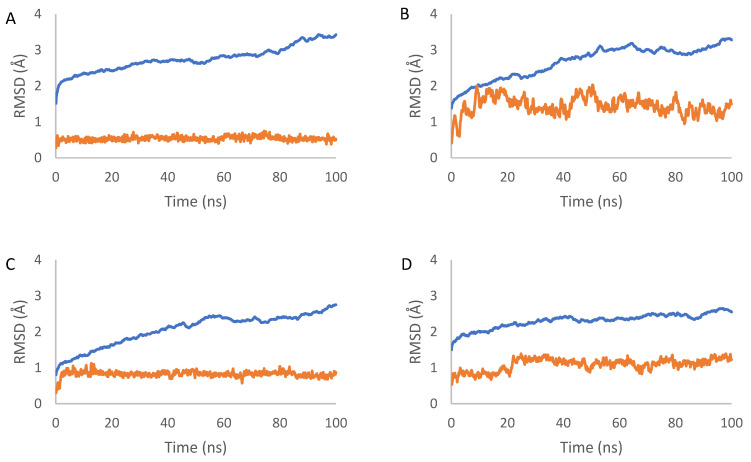

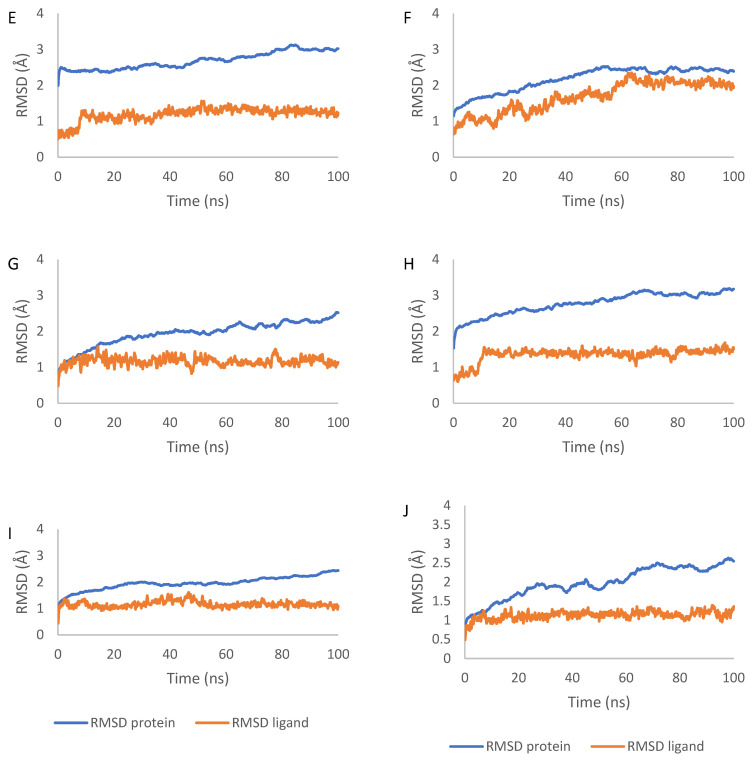
RMSD plot of the complexes 1–10 (**A**–**J**) in the MD simulation time. RMSD values of the proteins are depicted in blue, and the ligands are exhibited in orange.

RMSD values of the protein backbone plateaued around 2.4 Å to 3.4 Å after approximately 20 to 60 ns, depending on the protein. The RMSD values of the proteins fluctuated between 0.15 nm and 0.34 nm for complex 1 (average of 0.28 nm), between 0.13 nm and 0.33 nm for complex 2 (average of 0.27 nm), between 0.08 nm and 0.28 nm for complex 3 (average of 0.21 nm), between 0.15 nm and 0.27 nm for complex 4 (average of 0.23 nm), between 0.20 nm and 0.31 nm for complex 5 (average of 0.27 nm), between 0.12 nm and 0.25 nm for complex 6 (average of 0.19 nm), between 0.08 nm and 0.25 nm for complex 7 (average of 0.19 nm), between 0.15 nm and 0.32 nm for complex 8 (average of 0.28 nm), between 0.09 nm and 0.24 nm for complex 9 (average of 0.20 nm), and between 0.08 nm and 0.26 nm for complex 10 (average of 0.20 nm). Structural fluctuations were less than 0.3 nm, suggesting that the complex attained stable equilibrium (Figure 5).

The RMSD of the ligands increased from the start of the simulation to around 60 ns, after which it remained relatively stable with very low variation until the end of the simulation. The RMSD values of the ligands fluctuated between 0.03 nm and 0.08 nm for complex 1 (average of 0.05 nm), between 0.04 nm and 0.20 nm for complex 2 (average of 0.14 nm), between 0.03 nm and 0.11 nm for complex 3 (average of 0.08 nm), between 0.05 nm and 0.14 nm for complex 4 (average of 0.11 nm), between 0.05 nm and 0.16 nm for complex 5 (average of 0.12 nm), between 0.06 nm and 0.24 nm for complex 6 (average of 0.17 nm), between 0.05 nm and 0.16 nm for complex 7 (average of 0.12 nm), between 0.06 nm and 0.17 nm for complex 8 (average of 0.13 nm), between 0.04 nm and 0.16 nm for complex 9 (average of 0.12 nm), and between 0.05 nm and 0.14 nm for complex 10 (average of 0.1 nm). These values, less than 0.15 nm, indicate the stability of the docking interaction poses (Figure 5).

The 100 ns MD simulations showed that the ligand remained stably bound within the binding pocket of the protein over the course of the trajectory.

### 2.8. Network Pharmacology Results

**Figure 6:**
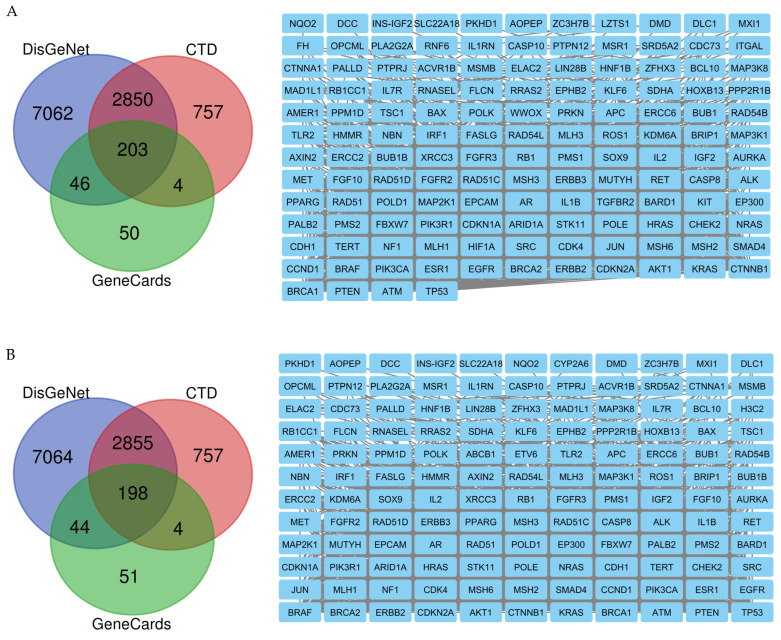
(**A**) Compound **1**- and (**B**) compounds **2**–**6**-associated targets of cancers, zoomed in to show the specific targets.

Androgen receptor (AR), hypoxia-inducible factor 1-alpha (HIF1A), and integrin alpha-L (ITGAL) were the top-ranking targets for compound **1** identified via BindingDB, DrugBank, ChEMBL, and SwissTargetPrediction. Meanwhile, the main target for compounds **2**–**6** was P-glycoprotein 1 (ABCB1). Using the keywords ‘neoplasms’ and ‘cancer’, 10,161 potential neoplasms targets were retrieved from the DisGeNet database, 3814 neoplasms targets were retrieved from the CTD database for all compounds, and 305 and 298 cancer targets were retrieved from the GeneCards database for compounds **1** and **2**–**6**, respectively. A total of 203 and 199 cancer targets were generated from the intersection of these three databases using a Venn diagram for compounds **1** and **2**–**6**, respectively (Figure 6).

Proteins interact with each other to participate in diverse biological processes such as biological signaling, regulation of gene expression, energy and material metabolism, and cell cycle regulation. In this study, protein–protein interaction (PPI) analysis between the compounds and associated cancer targets was performed to elucidate the underlying mechanisms using STRING.

**Figure 7:**
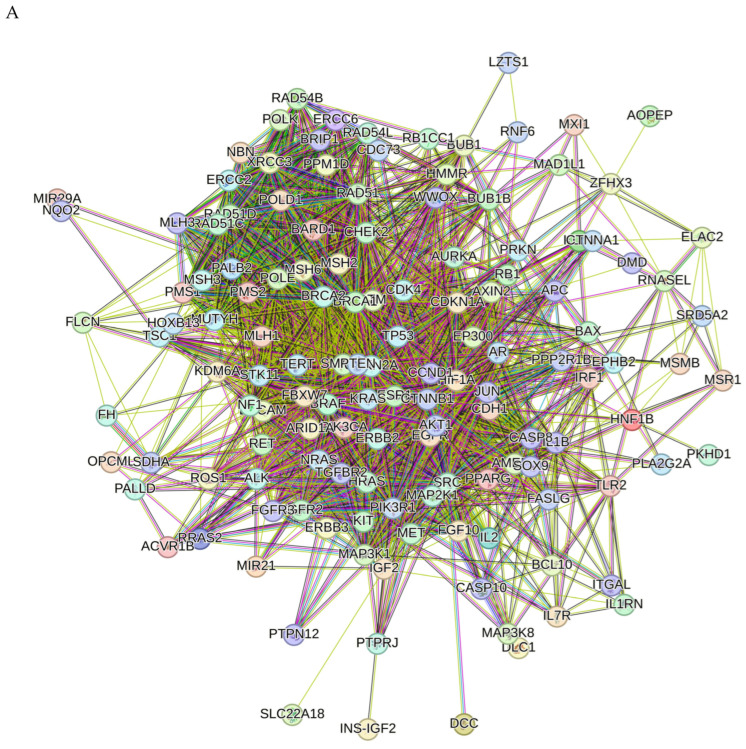

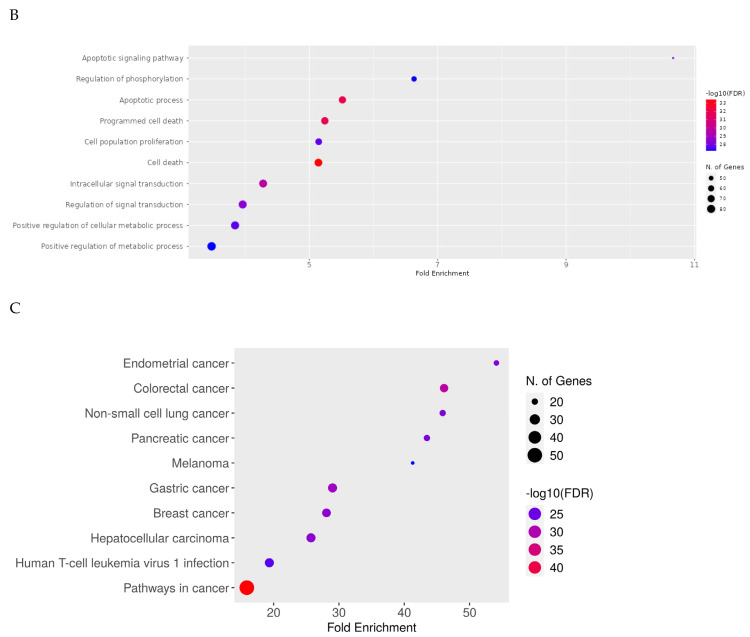
PPI network of 203 compound **1**-associated targets of cancers (**A**); GO analysis of all the targets (**B**); and KEGG analysis of all the targets (**C**).

The PPI network for compound **1** is visualized in Figure 7A. Importantly, *TP53*, *ATM*, *PTEN*, *CTNNB1*, *BRCA1*, *KRAS*, *AKT1*, *CDKN2A*, *BRCA2*, and *ERBB2* were identified as core compound **1**-associated targets in cancer. GO analysis of target genes of cancer targets was related to the regulation of the apoptotic signaling pathway and many other pathways (Figure 7B). According to KEGG analysis, most of the target genes were involved in cancer-related pathways (Figure 7C).

**Figure 8:**
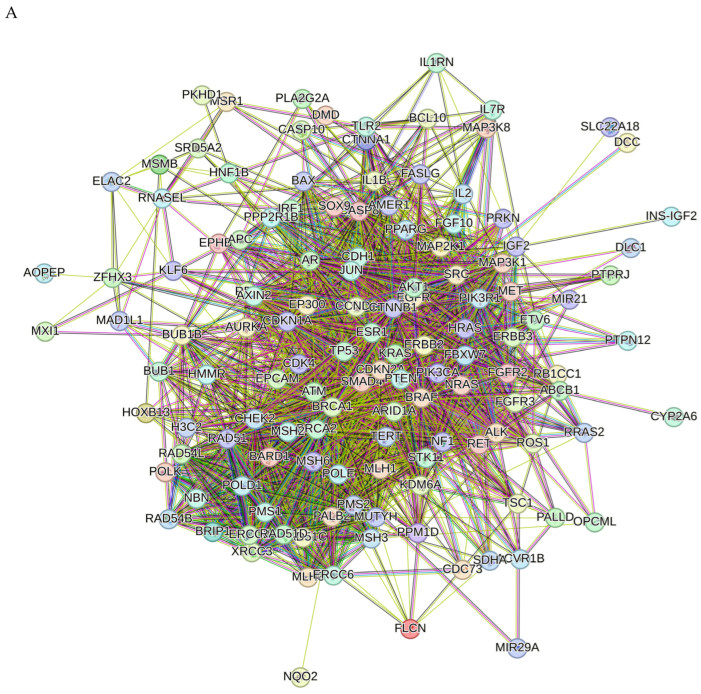

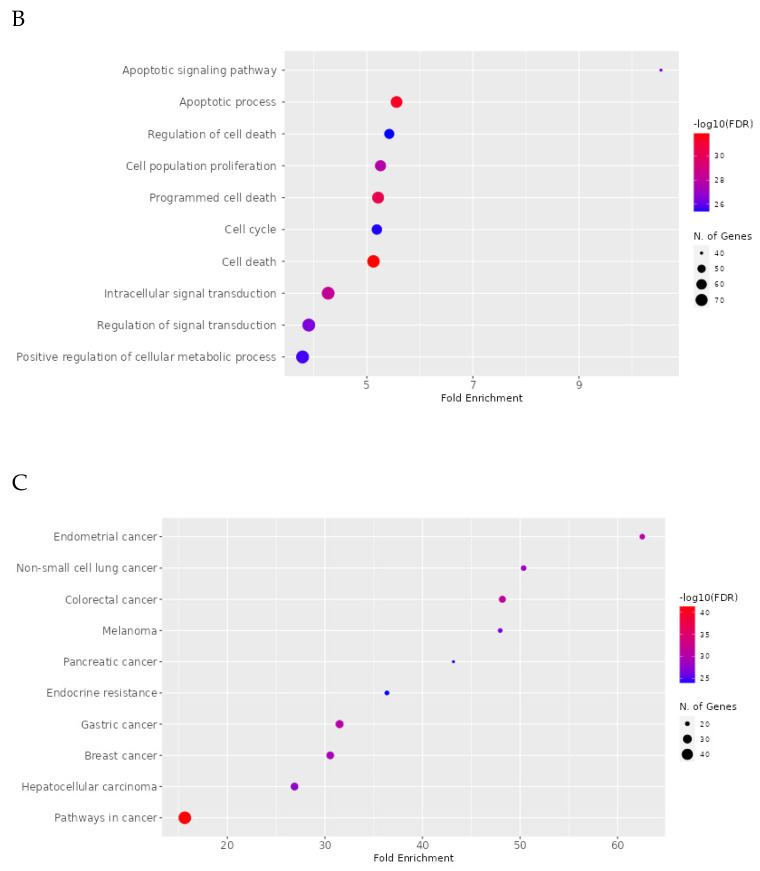
PPI network of 199 compounds **2**–**6**-associated targets of cancers (**A**); GO analysis of all the targets (**B**); and KEGG analysis of all the targets (**C**).

The PPI network for compounds **2**–**6** is visualized in Figure 8A. Importantly, *TP53*, *PTEN*, *ATM*, *BRCA1*, *KRAS*, *CTNNB1*, *AKT*, *CDKN2A*, *ERBB2*, and *BRCA2* were identified as core compounds **2**–**5**-associated targets in cancer. GO analysis of target genes of cancer targets was related to the regulation of the apoptotic signaling pathway and many other pathways (Figure 8B). According to KEGG analysis, most of the target genes were involved in cancer-related pathways (Figure 8C).

## 3. Discussion

Plants constitute a rich reservoir of structurally diverse chemical entities classified as secondary metabolites. These encompass several classes of compounds, including alkaloids, phenolic compounds, and terpenoids, among others, biosynthesized by plants via various biochemical pathways. Although over 2,140,000 secondary metabolites of elucidated structures have been documented in the plant kingdom, this number continues to rise rapidly [28,29]. The structural diversity of secondary plant metabolites provides an extensive chemical potential for the identification of novel lead compounds with therapeutic properties. Moreover, numerous natural compounds may have served as a scaffold for semi-synthetic drug analogs exhibiting improved pharmacological attributes [30,31].

In the era of modern drug development, computational and experimental approaches converge to harness the potential of plant secondary metabolites. Virtual screening, molecular modeling, and other in silico methodologies play pivotal roles in identifying lead compounds, predicting their interactions with biological targets, and optimizing their pharmacological profiles. Integrating these computational techniques with traditional experimental methods expedites the identification of novel drug candidates, paving the way for the development of innovative therapies [32,33,34].

The current research has focused on the compound 7α-acetoxy-6β-hydroxyroyleanone (Roy) isolated from the *P. grandidentatus*; it belongs to the broader class of abietanes that encompasses a diverse array of plant-derived diterpenoid compounds demonstrating promising biological effects, including antibacterial, anti-inflammatory, and immunomodulatory properties, as well as cytotoxic activities in numerous cancer cell lines [35,36,37,38,39,40].

Roy shows antibacterial efficacy against methicillin-resistant *Staphylococcus aureus*, vancomycin-resistant *Enterococcus faecalis*, or multidrug-resistant *Mycobacterium tuberculosis*. It exhibits even better activity than some of the reference drugs currently used [35,36,37]. Notably, Roy did not demonstrate synergistic interactions with other cell wall-active antibiotics like methicillin, vancomycin, and ampicillin. Roy also did not directly affect cell membrane integrity. However, scanning electron micrographs revealed aberrations in cell morphology, including cell clustering, wall disruption, and loss of regular spherical shape. An analysis of peptidoglycan composition indicated a relative accumulation of substructures containing truncated glycine bridges between muropeptide subunits upon Roy treatment. Overall, while the exact mechanism remains unclear, the data indicate that cell wall biosynthesis is a likely target for the antibacterial action of Roy [41].

Roy potently inhibits 5-lipoxygenase (5-LO) activity, which is a key enzyme in leukotriene biosynthesis. Leukotrienes are pro-inflammatory lipid mediators involved in inflammatory disorders. Specifically, this compound suppresses 5-LO product formation in cell-free assays with an IC_50_ of 1.3 μg/mL and in human neutrophils with an IC_50_ of 5.1 μg/mL. Therefore, the inhibition of 5-LO and leukotriene production appears to be an anti-inflammatory mode of action of 7α-acetoxy-6β-hydroxyroyleanone [38]. Roy also displays immunosuppressive activity by inhibiting lymphocyte proliferation. When human lymphocytes are stimulated with mitogens, this compound dose-dependently inhibits their proliferation with higher potency than other tested abietane diterpenoids [39].

Roy showed promising cytotoxic activity against several human cancer cell lines, including leukemia (CCRF-CEM), lung adenocarcinoma (A549), breast cancer (MDA-MB-231, MCF-7, SkBr3), colon cancer (HCT116), and glioblastoma (U87, A172, U118, U373) cells. This compound has consistently demonstrated strong antiproliferative, cytotoxic, and anticancer activities across cell lines from various tumor types, including aggressive and multidrug-resistant cancers. Its ability to selectively target cancer cells, interact with cancer-related proteins like PKCs, and accumulate intracellularly highlights its potential for development into an anticancer therapeutic lead [40,42,43,44].

The focus of this work has been on the synthesis of five Roy derivatives to assess their chemical properties through in silico methods. Computational tools aimed to identify new derivatives with even more favorable biological characteristics. This integrated approach, combining experimental results with computational structural analysis, represents a significant advancement in the search for effective compounds with potential therapeutic applications.

The ADMET and drug-likeness analysis using SwissADME revealed generally favorable pharmacokinetic parameters for Roy (compound **1**) and the semi-synthetic derivatives (compounds **2**–**6**). The majority of the compounds exhibited acceptable molecular weights, lipophilicity, aqueous solubility, and predicted gastrointestinal absorption. However, compounds **2**, **3**, and **6** slightly deviated from Lipinski’s rule of five, with molecular weights exceeding 500 Da and having higher lipophilicity (consensus log P > 5). These findings suggest that while most of the derivatives possess drug-like properties, some structural modifications, such as the introduction of bulky aromatic substituents, may impact their physicochemical characteristics and require further optimization to enhance their drug-likeness.

Furthermore, the predictions of toxicity indicated low risks of acute oral toxicity, placing semi-synthetic derivatives in category III, with LD_50_ ranging from 50 to 300 mg/kg. No evidence of hepatotoxicity was found, along with a minimal probability of mutagenicity and cytotoxicity.

The analysis of antineoplastic and anticarcinogenic activities using the PASS algorithm showed strong predicted anticancer potential across all agents, with Pa values between 0.819 and 0.879, surpassing the 0.3 activity threshold. Compound **1** (Roy) exhibited the highest predicted antineoplastic activity with a Pa value of 0.879. Comparing the PASS results of the semi-synthetic derivatives (compounds **2**–**6**) with the parent compound Roy (compound **1**), we observed that the introduction of certain functional groups, such as the phenylacetyl moiety in compound **2** and the 2-naphthoate group in compound **4**, led to slightly lower predicted antineoplastic activity (Pa values of 0.819 and 0.822, respectively) compared to Roy (Pa = 0.879). On the other hand, the presence of the (4-methyl)benzoyl group in compound 3 and the butanoyl group in compound 5 resulted in comparable anticancer potential to Roy, with Pa values of 0.834 and 0.858, respectively.

The DFT computations revealed that strategic structural modifications can modulate the electronic properties and reactivity profiles of Roy (compound **1**) and analogs (compounds **2**–**6**). The calculations revealed that the structural modifications influenced the electronic parameters of the compounds. Compound **2** exhibited the lowest HOMO–LUMO gap (3.39 eV) and the highest softness (0.29 eV^−1^), suggesting higher chemical reactivity compared to the other derivatives. Conversely, compound 6 had the largest HOMO–LUMO gap (3.79 eV) and the lowest softness (0.26 eV^−1^), indicating higher stability. These findings highlight the impact of specific structural features on the electronic characteristics of the compounds. The presence of the phenylacetyl moiety in compound **2** seems to contribute to its increased reactivity, while the benzoyloxy and acetoxy substituents in compound 6 appear to enhance its stability. Understanding these structure–property relationships is crucial for the rational design and optimization of Roy-based derivatives with desired electronic attributes and reactivity profiles.

Molecular docking uncovered favorable binding against pivotal pro- and anti-apoptotic regulators, cell cycle proteins, receptor tyrosine kinases, and DNA repair factors. The molecular docking results revealed that different structural modifications could influence the binding preferences of the compounds toward specific protein targets. For instance, compound **2** exhibited the strongest binding affinity to BCL-2 (−10.46 kcal/mol), while compound **4** showed the highest binding energies for EGFR (−11.65 kcal/mol), BCL-XL (−12.84 kcal/mol), caspase 3 (−11.86 kcal/mol), CDK2 (−13.49 kcal/mol), VEGFR (−12.39 kcal/mol), and p53 (−11.40 kcal/mol). These findings suggest that the introduction of specific substituents can direct the compounds’ preferences toward particular molecular targets involved in cancer pathways.

Additionally, the network pharmacology approaches provided useful insights into the potential mechanisms of action of the compounds. The construction of protein–protein interaction networks revealed crucial nodes across signaling cascades related to apoptosis, cell proliferation, angiogenesis, and DNA repair. Enrichment analysis indicated the involvement of cancer-associated pathways. Importantly, the integration of target prediction and network analysis facilitated the mapping of key biological processes and pathways that could be affected by the compounds to exert their anticancer effects.

The lowest HOMO–LUMO gap and the greatest polarizability exhibited by compound **2** suggest that it has higher chemical reactivity and lower kinetic stability compared to the other investigated compounds. This finding can be related to the molecular docking results, where compound **2** demonstrated strong binding affinity towards the BCL-2 protein, with a binding energy of −10.46 kcal/mol. The enhanced reactivity of compound **2**, as indicated by its electronic properties, may contribute to its potential interactions with biological targets.

To explore the correlation between DFT descriptors and the predicted anticancer activity, we analyzed the relationships between the PASS results (Pa values) and the calculated electronic parameters. However, no clear correlations were observed between the HOMO–LUMO gap, ionization energy, electron affinity, electronegativity, chemical potential, global chemical hardness, global chemical softness, global electrophilicity index, or maximum additional electric charge and the predicted antineoplastic activity of compounds **1**–**6**. This suggests that the anticancer potential of these derivatives may be influenced by a complex interplay of factors beyond the individual electronic descriptors.

Furthermore, we investigated the potential links between DFT parameters and ADMET properties, such as lipophilicity, water solubility, and plasma protein binding. Again, no strong correlations were identified, indicating that the electronic characteristics alone may not be sufficient predictors of the pharmacokinetic behavior of these compounds.

Despite the lack of clear correlations, the information gained from DFT calculations can still guide the rational design and optimization of compounds for desired biological activity and pharmacokinetic properties. By combining insights from various computational methods, including DFT, molecular docking, and ADMET predictions, we can gain a more comprehensive understanding of the therapeutic potential of the studied compounds. Further experimental investigations are necessary to validate these computational findings and unravel the complex relationships between electronic structure, molecular interactions, and pharmacological properties.

This concerted computational investigation of Roy and its derivatives substantiates their potential as anticancer compounds.

## 4. Materials and Methods

### 4.1. Compounds

#### 4.1.1. General Experimental Procedures

Nuclear magnetic resonance (NMR) spectra were obtained using a 300 MHz Bruker Fourier spectrometer and a 400 MHz Bruker Fourier spectrometer (Bruker, Mannheim, Germany). Melting point determinations were performed on a Stuart Scientific SMP10 (Merck KGaA, Darmstadt, Germany) model with 230 V AC/DC capability. Optical rotation measurements were carried out in chloroform solutions utilizing an Anton Paar MC100 polarimeter (Anton Paar, Graz, Austria). Attenuated total reflectance Fourier transform infrared (ATR-FTIR) spectra were recorded on a PerkinElmer Spectrum Two infrared spectrophotometer equipped with a Universal ATR. Liquid chromatography-high resolution mass spectrometry (LC-HRMS) data were acquired using a Dionex Ultimate 3000 ultra high-performance liquid chromatograph (UHPLC) system (Thermo Fisher Scientific, Waltham, MA, USA) coupled to a Thermo Scientific Q Exactive hybrid quadrupole-Orbitrap mass analyzer. The UHPLC component consisted of a multiple wavelength detector and an imChem Surf C18 TriF reverse phase column, applying a 10 min linear solvent gradient of 20–30% aqueous acetonitrile at a 0.2 mL/min flow rate alongside 250 nm UV detection. All solvents were distilled from commercial-grade sources. Pyridine and benzoyl/anhydride reagents were used without previous purification.

#### 4.1.2. Plant Material

The plant material, from *Plectranthus grandidentatus* Gürke, utilized in this study was obtained from the Parque Botânico da Tapada da Ajuda (Instituto Superior Agrário, Lisbon, Portugal) based on cuttings acquired from the Kirstenbosch National Botanical Garden (Cape Town, South Africa). Herbarium voucher specimens were deposited in the João de Carvalho e Vasconcellos Herbarium (ISA) under accession number 841/2007. Following collection between 2007 and 2008, the *P. grandidentatus* plant materials were dried and stored at room temperature and protected from light and moisture exposure. Taxonomic verification was conducted by cross-referencing The Plant List (http://www.theplantlist.org, accessed on 8 September 2023) and World Flora Online databases (http://www.worldfloraonline.org/, accessed on 8 September 2023).

#### 4.1.3. Extraction and Isolation

An ultrasound-assisted acetone extraction protocol adapted from Bernardes et al. [45] was implemented on dried aerial *P. grandidentatus* material harvested at room temperature. Crude extracts were obtained via sonication for 30-min cycles in an ultrasonic bath (Sonorex Super RK 510 H; Bandelin) operating at 35 Hz with a maximum power input of 320 W. The plant materials underwent three sequential extractions prior to filtration and solvent evaporation under vacuum at 40 °C, producing 2.3% *w*/*w* extracted yield. These *P. grandidentatus* extracts were subsequently fractionated using sequential chromatographic separation procedures. Ultimately, compound **1** (7α-acetoxy-6β-hydroxyroyleanone (Roy)) was isolated as yellow crystal plates via recrystallization from n-hexane. Structural characterization data aligned with prior reports [46,47,48].

*7α-acetoxy-6β-hydroxyroyleanone (Roy)* (**1**): 1H-NMR (300 MHz, Chloroform-*d*, ppm): δ 7.22 (s, 1H, 12-OH), 5.66 (dd, *J* = 2.2, 0.7 Hz, 1H, H-7β), 4.31 (s, 1H, H-6α), 3.16 (sept, *J* = 7.1 Hz, 1H, H-15), 2.63 (d, *J* = 12.8 Hz, 1H, H-1β), 2.04 (s, 3H, 7α-OAc), 1.89–1.78 (m, 1H, H-2β), 1.61 (s, 3H, Me-20), 1.55–1.46* (m, 2H, H-2α and H-3β), 1.33 (s, 1H, H-5α), 1.23* (s, 3H, Me-19), 1.22* (d, *J* = 7.1 Hz, 3H, Me-17), 1.21* (s, 1H, H-3α ^+^), 1.20* (d, *J* = 7.1 Hz, 3H Me-16), 1.18* (s, 1H H-1α ^+^), 0.94 (s, 3H, Me-18). *Overlapped signals, ^+^Can be changed.

Semi-synthesis of derivatives of compound **1**

General procedure for semi-synthesis of ester derivatives

**Figure 9:**
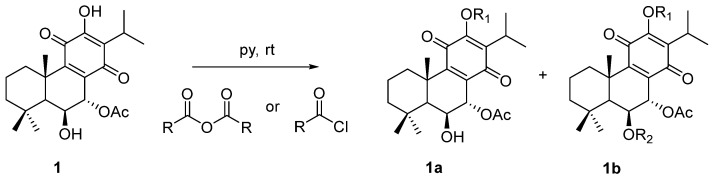
Reaction of semi-synthesis of Roy **1** ester derivatives.

The general esterification procedure (Figure 9) was carried out following the optimized conditions described by Isca et al. [49]. Compound **1** (20 μmol, 1 equiv.) was dissolved in pyridine (0.5 mL) under inert conditions prior to the addition of the corresponding benzoyl chloride or acetic anhydride (3–21 equiv.). Reactions proceeded at room temperature under inert conditions, with stirring, until the complete conversion of compound **1**. The crude reaction mixture underwent preparative chromatography for purification.

*7α-acetoxy-6β-hydroxy-12-O-phenylacetyl-royleanone* (**2**): The compound was prepared according to the general procedure, with phenylacetyl chloride (422.6 μmol, 10 equiv.) and then let to react for 60 min. The crude mixture was purified by preparative chromatography with a mixture of dichloromethane/acetone (99:1). The pure product (16%) was obtained as a dark yellow oil. ${\left[\alpha \right]}_{D}^{20}=+20.0^{{}^{\circ}}$ (*c* 0.200, CHCl_3_). IR ${\bar{\nu }}_{\max}$: 3516.3, 2965.8, 2937.5, 2872.7, 1771.8, 1727.3, 1670.6, 1456.0, 1225.4, 1136.3, 1104.0, 1027.0, 751.8, 723.5, 703.2 cm^−1^. ^1^H NMR (400 MHz, Chloroform-*d*, ppm): δ 7.37 (d, *J* = 4.2 Hz, 4H, H-2′, H-3′), 7.31 (dt, *J* = 8.5, 4.2 Hz, 1H, H-4′), 5.63 (s, 1H, H-7β), 4.31 (s, 1H, H-6α), 3.93 (s, 2H, 12-COCH_2_), 2.99 (qui, *J* = 7.1 Hz, 1H, H-15), 2.51 (br d, *J* = 12.6 Hz, 1H, H-1β), 2.04 (s, 3H, 7α-OAc), 1.91–1.75 (m, 1H, H-2β), 1.63 (s, 3H, Me-20), 1.58–1.54 (m, 1H, H-2α), 1.46 (d, *J* = 13.1 Hz, 1H, H-3β), 1.32 (s, 1H, H-5α), 1.22* (s, 5H, Me-19, H-3α, H-1α), 1.04* (d, *J* = 7.1 Hz, 6H, Me-16, Me-17), 0.93 (s, 3H, Me-18). *Overlapped signal. ^13^C NMR (101 MHz, Chloroform-*d*, ppm): δ 185.86 (C-14), 179.69 (C-11), 169.82 (7α-COCH_3_), 168.94 (12-COCH_2_), 153.05 (C-9), 149.59 (C-12), 135.67 (C-8), 132.65 (C-1′), 129.68 (C-2′ ^+^), 128.88 (C-3′ ^+^), 127.69 (C-4′), 68.98 (C-7), 67.36 (C-6), 49.89 (C-5), 42.40 (C-3), 40.89 (12-COCH_2_), 39.00 (C-10), 38.45 (C-1), 33.86 (C-4), 33.65 (C-18), 25.05 (C-15), 23.99 (C-19), 21.85 (C-20), 21.04 (7α-COCH_3_), 20.18 (C-17), 20.04 (C-16), 19.03 (C-2). ^+^Can be changed. HRMS (ESI-MS): *m*/*z* calculated for C_30_H_37_ClO_7_ [2M + Na]^+^ 1039.4814, found 1039.48210.

*7α-acetoxy-6β-hydroxy-12-O-(4-methyl)benzoylroyleanone* (**3**): The compound was prepared according to the general procedure, with 4-toluoylbenzoyl chloride (88.4 μmol, 3 equiv.) and then let to react for 30 min. The crude mixture was purified by preparative chromatography with a mixture of dichloromethane/ethyl acetate (97:3). The pure product (53%) was obtained as a yellow amorphous powder. mp: 226–228 °C. ${\left[\alpha \right]}_{D}^{20}=+46.4^{{}^{\circ}}$ (*c* 0.280, CHCl_3_). IR ${\bar{\nu }}_{\max}$: 3551.8, 2961.7, 2929.1, 2870.5, 1739.3, 1726.3, 1667.6, 1648.0, 1609.0, 1609.0, 1465.5, 1374.2, 1250.3, 1224.3, 1178.6, 1139.5, 1100.4, 1067.8, 1009.1, 963.5, 898.3, 836.3, 823.3, 741.8, 686.4 cm^−1^. ^1^H NMR (400 MHz, Chloroform-*d*, ppm): δ 8.04 (d, *J* = 8.3 Hz, 2H, H-2′) ^+^, 7.32 (d, *J* = 8.0 Hz, 2H, H-3′) ^+^, 5.69 (d, *J* = 2.0 Hz, 1H, H-7β), 4.34 (s, 1H, H-6α), 3.19 (hept, *J* = 7.1 Hz, 1H, H-15), 2.52–2.50 (m, 1H, H-1β), 2.45 (s, 3H, Me-7′), 2.07 (s, 3H, 7α-OAc), 1.80 (dt, *J* = 13.7, 3.6 Hz, 1H, H-2β), 1.63 (s, 3H, Me-20), 1.55 (dt, *J* = 14.1, 3.7 Hz, 1H, H-2α), 1.46 (dt, *J* = 12.8, 3.4 Hz, 1H, H-3β), 1.37 (s, 1H, H-5α), 1.25–1.19* (m, 11H, Me-19, Me-17, H-3α, Me-16, H-1α), 0.95 (s, 3H, Me-18). *Overlapped signal. ^13^C NMR (101 MHz, Chloroform-*d*, ppm): δ 186.00 (C-14), 179.94 (C-11), 169.87 (7α-COCH_3_), 164.19 (12-COO), 153.20 (C-9), 149.98 (C-12), 145.37 (C-4′), 139.61 (C-13), 135.71 (C-8), 130.72 (C-3′ ^+^), 129.63 (C-2′ ^+^), 125.39 (C-1′), 69.07 (C-7), 67.41 (C-6), 49.93 (C-5), 42.45 (C-3), 39.04 (C-10), 38.47 (C-1), 33.87 (C-4), 33.67 (C-18), 25.26 (C-15), 24.00 (C-19), 21.98 (C-5′), 21.91 (C-20), 21.07 (7α-COCH_3_), 20.58 (C-16), 20.35 (C-17), 19.03 (C-2). ^+^Can be changed. HRMS (ESI-MS): *m*/*z* calculated for C_30_H_36_O_7_ [M + H]^+^ 509.2534, found 509.25375.

*7α-acetoxy-6β-hydroxy-12-O-(2-naphtoate)benzoylroyleanone* (**4**): The compound was prepared according to the general procedure, with 2-Naphthoyl chloride (384.2 μmol, 10 equiv.) and then let to react overnight. The crude mixture was purified by preparative chromatography with a mixture of dichloromethane/acetone (99:1). The pure product (68%) was obtained as a yellow amorphous powder. mp: 230–232 °C. ${\left[\alpha \right]}_{D}^{20}=+53.9^{{}^{\circ}}$ (*c* 0.167, CHCl_3_). IR ${\bar{\nu }}_{\max}$: 3467.0, 2965.0, 2922.6, 2854.2, 1745.8, 1736.0, 1657.8, 1631.7, 1609.0, 1576.3, 1459.0, 1367.7, 1276.4, 1250.3, 1217.7, 1185.1, 1142.8, 1123.2, 1097.1, 1061.3, 1022.1, 1009.1, 829.8, 777.7, 761.4, 732.0 cm^−1^. ^1^H NMR (400 MHz, Chloroform-*d*): δ 8.75 (d, *J* = 1.7 Hz, 1H, H-1′ ^+^), 8.13 (dd, *J* = 8.5, 1.7 Hz, 1H, H-3′ ^+^), 8.00 (d, *J* = 8.1 Hz, 1H, H-4′), 7.96 (d, *J* = 8.7 Hz, 1H, H-8′ ^++^), 7.93 (d, *J* = 8.0 Hz, 1H, H-5′ ^++^), 7.65 (t, *J* = 7.4 Hz, 1H H-6′ ^+++^), 7.59 (t, *J* = 7.5 Hz, 1H, H-7′ ^+++^), 5.70 (d, *J* = 2.0 Hz, 1H, H-7β), 4.35 (s, 1H, H-6α), 3.23 (qui, *J* = 7.1 Hz, 1H, H-15), 2.52 (br s, 1H, H-1β), 2.09 (s, 3H, 7α-OAc), 1.89–1.74 (m, 1H, H-2β), 1.65 (s, 3H, Me-20), 1.55 (dt, *J* = 14.1, 3.6 Hz, 1H, H-2α), 1.47 (d, *J* = 13.2 Hz, 1H, H-3β), 1.39 (s, 1H, H-5α), 1.24* (d, *J* = 7.1 Hz, 11H, Me-19, Me-17, H-3α, Me-16, H-1α), 0.96 (s, 3H, Me-18). *Overlapped signal; ^+^, ^++^, and ^+++^Can be changed. ^13^C NMR (101 MHz, Chloroform-*d*, ppm): δ 185.98 (C-14), 179.90 (C-11), 169.87 (7α-COCH_3_), 164.44 (12-COO), 153.18 (C-9), 150.00 (C-12), 136.22 (C-4a), 132.76 (C-8a), 132.58 (C-1′ ^+^), 129.73 (C-4′ ^++^), 129.15 (C-6′ ^+++^), 128.81 (C-8′ ^++^), 128.02 (C-5′ ^++^), 127.15 (C-7′ ^+++^), 125.55 (C-3′ ^+^), 125.29 (C-2′), 69.06 (C-7), 67.42 (C-6), 49.93 (C-5), 42.44 (C-3), 39.07 (C-10), 38.49 (C-1), 33.88 (C-4), 25.34 (C-15), 24.00 (C-19), 21.92 (C-20), 21.09 (7α-COCH_3_), 20.65 (C-16), 20.39 (C-17), 19.03 (C-2). ^+^, ^++^, and ^+++^Can be changed. HRMS (ESI-MS): *m*/*z* calculated for C_33_H_36_O_7_ [M + H]^+^ 545.2534, found 545.25428.

*7α-acetoxy-6β-hydroxy-12-O-butanoylroyleanone* (**5**): The compound was prepared according to the general procedure, with butyryl chloride (181.3 μmol, 6 equiv.) and then let to react for 5 min. The crude mixture was purified by preparative chromatography with dichloromethane. The pure product (94%) was obtained as an amber amorphous powder. mp: 148–150 °C. ${\left[\alpha \right]}_{D}^{20}=+54.2^{{}^{\circ}}$ (*c* 0.241, CHCl_3_). IR ${\bar{\nu }}_{\max}$: 3516.0, 2965.0, 2932.4, 2877.0, 1768.6, 1742.6, 1732.8, 1670.9, 1657.8, 1612.2, 1465.5, 1367.7, 1276.4, 1224.3, 741.8 cm^−1^. ^1^H NMR (400 MHz, Chloroform-*d*, ppm): δ 5.65 (d, *J* = 2.0 Hz, 1H, H-7β), 4.31 (s, 1H, H-6α), 3.10 (p, *J* = 7.1 Hz, 1H, H-15), 2.59 (q, *J* = 7.4 Hz, 2H, H-1′), 2.49 (d, *J* = 12.9 Hz, 1H, H-1β), 2.05 (s, 3H, 7α-OAc), 1.79* (q, *J* = 7.4 Hz, 3H, H-2β, H-2′), 1.62 (s, 1H, Me-20), 1.55 (dq, *J* = 14.3, 3.7 Hz, 1H, H-2α), 1.45 (dt, *J* = 13.5, 3.3 Hz, 1H, H-3β), 1.33 (s, 1H, H-5α), 1.23 (d, *J* = 11.4 Hz, 4H, Me-19, H-3α ^+^), 1.18* (dd, *J* = 7.1, 3.1 Hz, 7H, Me-16, Me-17, H-1α ^+^), 1.05 (t, *J* = 7.4 Hz, 1H, Me-3′), 0.93 (s, 3H, Me-18). *Overlapped signal; ^+^Can be changed. ^13^C NMR (101 MHz, Chloroform-*d*, ppm): δ 185.98 (C-14), 179.93 (C-11), 171.10 (12-COO), 169.88 (7α-COCH_3_), 153.06 (C-9), 149.65 (C-12), 139.30 (C-13), 135.69 (C-8), 69.03 (C-7), 67.32 (C-6), 49.90 (C-5), 42.43 (C-3), 38.98 (C-10), 38.43 (C-1), 35.81 (C-1′), 33.85 (C-4), 33.65 (C-18), 25.25 (C-15), 23.97 (C-19), 21.84 (C-20), 21.05 (7α-COCH_3_), 20.41 (C-16), 20.33 (C-17), 19.04 (C-2), 18.36 (C-2′), 13.77 (C-3′). HRMS (ESI-MS): *m*/*z* calculated for C_26_H_36_O_7_ [M + H]^+^ 461.2534, found 461.25346.

The synthesis of compound **6** (7α-acetoxy-6β-benzoyloxy-12-O-benzoylroyleanone) was presented previously [47].

### 4.2. ADMET and Drug-Likeness Analysis

The prediction of absorption, distribution, metabolism, excretion, and toxicity (ADMET) parameters and drug-like properties of the compounds was performed using the SwissADME server (http://www.swissadme.ch/, accessed on 6 September 2023). Key physicochemical parameters calculated encompassed molecular weight (MW), atom counts, molecular refractivity (MR), and polar surface area (PSA). Lipophilicity was evaluated via iLOGP, XLOGP3, WLOGP, MLOGP, and SILICOS-IT models to generate log P octanol/water (log Po/w) values. Aqueous solubility (log S) values were predicted through ESOL, Ali, and SILICOS-IT models. The pharmacokinetic descriptors examined included gastrointestinal absorption (GI), blood–brain barrier (BBB) permeability, the substrate potential of P-glycoprotein (P-gp), as well as the inhibition of five major cytochrome P450 isoforms (CYP1A2, CYP2C19, CYP2C9, CYP2D6, and CYP3A4) and skin permeability (log Kp). Compound drug-likeness was assessed through established criteria, including Lipinski, Ghose, Veber, Egan, and Muegge filters. Additional calculations provided key medicinal chemistry descriptors [50,51].

### 4.3. Toxicity Prediction and Molecular Properties

The toxicity profiles of the compounds were evaluated using the Prediction of toxicity of chemicals (ProTox-II) server (http://tox.charite.de/protox_II, accessed on 20 September 2023), which predicts LD50 values (mg/kg body weight), hepatotoxicity, carcinogenicity, immunotoxicity, mutagenicity, cytotoxicity, effects on key cellular signaling pathways [aryl hydrocarbon receptor (AhR), androgen receptor (AR), aromatase, estrogen receptor α (Erα), and peroxisome proliferator-activated receptor γ (PPARγ)], and stress response pathways [nuclear factor (erythroid-derived 2)-like 2/antioxidant responsive element (nrf2/ARE), heat shock factor response element (HSE), mitochondrial membrane potential (MMP), phosphoprotein (tumor suppressor) p53, and ATPase family AAA domain-containing protein 5 (ATAD5)] [52]. Additionally, the StopTox server (https://stoptox.mml.unc.edu/, accessed on 20 September 2023) was utilized to predict the acute toxicity potential of these compounds [53]. The OSIRIS platform was also used to assess risks of irritation, mutagenicity, carcinogenicity, and adverse reproductive outcomes [54].

### 4.4. Anticarcinogenic Activity

Evaluation of prospective anticancer attributes of the selected compounds was performed using the Prediction of Activity Spectra for Substances (PASS) server (http://www.pharmaexpert.ru/passonline/, accessed on 25 October 2023). PASS is a tool used for the forecasting of diverse activities across various molecules. The predictive spectrum generated by PASS relies on the analysis of structural activity relationships (SARs) within the training dataset. The projected activity spectrum of a compound is presented as probable activity (Pa) or probable inactivity (Pi). Compounds exhibiting Pa > Pi are considered viable candidates for affirmative biological validation [55].

### 4.5. DFT Calculations

Ligand 3D structures were generated in Avogadro v1.95, followed by conversion in XYZ format. Density functional theory (DFT) computations used ORCA v5.03 software with the B3LYP density functional set and the Def2-TZVPP basis set, implementing a gas phase model [56]. The analysis of the DFT results was conducted by Avogadro [57].

### 4.6. Molecular Docking

#### 4.6.1. Protein and Ligand Preparation

The three-dimensional protein structures utilized in molecular docking evaluations were retrieved from the RCSB Protein Data Bank (PDB) (http://www.rcsb.org, accessed on 6 November 2023). Specifically, the structures of BCL-2 (PDB ID: 2W3L), BCL-XL (PDB ID: 3ZK6), caspase 3 (PDB ID: 1NMS), caspase 9 (PDB ID: 1NW9), CDK2 (PDB ID: 1DI8), CDK6 (PDB ID: 1XO2), EGFR (PDB ID: 1M17), VEGFR (PDB ID: 2OH4), p53 (PDB ID: 3DCY), and PARP1 (PDB ID: 5DS3) were retrieved. Proteins were prepared by removing heteroatoms and water molecules and adding missing atoms, polar hydrogens, and Kollman charges in AutoDock Tools v1.5.6. The optimized XYZ coordinate files generated for the ligand structures were utilized for the molecular docking simulations [58].

#### 4.6.2. Active Site Prediction

The Computed Atlas of Surface Topography of Proteins (CASTp) server (http://sts.bioe.uic.edu/castp/index.html?3trg, accessed on 6 November 2023) calculates the physicochemical and geometric properties of protein binding pockets and cavities that interact with small molecule ligands. For the protein targets under examination, CASTp was used to determine the active site location [59].

#### 4.6.3. Receptor–Ligand Docking

Molecular docking was performed in AutoDock with grid box dimensions defined for each protein target active site, with box dimensions of 60 × 60 × 60 points. Specifically, grid center coordinates denoted as (X, Y, Z) in angstroms encompassed BCL-2 (34.530, 31.076, and 1.645), BCL-XL (19.061, 51.294, and 0.154), caspase 3 (6.758, −0.081, and 9.017), caspase 9 (53.800, 18.030, and 92.471), CDK2 (−4.908, 47.554, and 14.485), CDK6 (29.145, 43.770, and 131.559), EGFR (30.025, 4.113, and 52.110), VEGFR (3.072, 38.530, and 15.593), and PARP-1 (−2.393, 39.874, and 13.085). A spacing of 0.5 Å between the grid points was applied. Docking used a Lamarckian genetic algorithm with 100 independent executions and a population size of 300 binding modes per ligand–receptor complex. Final binding poses were ranked by docking score, with the lowest energy conformer from each populated cluster selected as the representative for downstream 2D and 3D visual inspection of predicted intermolecular contacts using Discovery Studio [60].

### 4.7. Molecular Dynamics Simulations

Molecular dynamics (MD) simulations of selected ligand–protein complexes were performed utilizing NAMD software v2.14 [61], along with the CHARMM36m force field parameters [62] with topology files generated through CHARMM-GUI [63]. The docked protein–ligand complexes were solvated in a rectangular water box with an edge distance of 10 Å. Additionally, the Monte Carlo method was used to add KCl ions at a concentration of 0.15 M. Energy minimization was performed using the steepest descent method (5000 steps). Two stages of equilibration for 100 ps were carried out to bring the system to constant temperature (303.15 K) and pressure (1 atm). The first equilibration was performed in the NVT ensemble (constant number of particles, volume, and temperature), while the second was performed in the NPT ensemble (constant number of particles, pressure, and temperature). Finally, the MD simulation production step was conducted with a simulation time of 100 ns using an integration time of 2 fs. The VMD program [64] was used to retrieve the resulting simulation data, which included root mean square deviation (RMSD). The RMSD calculation used the coordinates after every 100 ps.

### 4.8. Network Pharmacology

#### 4.8.1. Identification of Potential Targets of Analyzed Compounds

Potential targets of analyzed compounds were identified and collected using the BindingDB database (https://www.bindingdb.org/bind/index.jsp, accessed on 5 February 2024), DrugBank (https://go.drugbank.com/, accessed on 5 February 2024), ChEMBL (https://www.ebi.ac.uk/chembl/, accessed on 5 February 2024), SwissTargetPrediction (http://www.swisstargetprediction.ch/, accessed on 5 February 2024).

#### 4.8.2. Associated Targets of Cancer Diseases

DisGeNet (https://www.disgenet.org/, accessed on 5 February 2024), the comparative toxicogenomics database, CTD (https://ctdbase.org/, accessed on 5 February 2024), and the human gene database, GeneCards (https://www.genecards.org/, accessed on 5 February 2024), were used to identify cancer targets with keywords ‘neoplasms’ and ‘cancer’. To identify connections between cancer target genes and active components, the online application Venny v2.1.0 (https://bioinfogp.cnb.csic.es/tools/venny/, accessed on 5 February 2024) was used.

#### 4.8.3. Visualization and Analysis of the Network of the Protein–Protein Interactions

In order to examine protein–protein interactions (PPI), the STRING platform (https://string-db.org/, accessed on 6 February 2024) was used to obtain human datasets with a confidence score exceeding 0.4. The resulting interaction network was then analyzed using Cytoscape v3.10.1 [65].

#### 4.8.4. Enrichment Analysis

The biological process, molecular function, and cellular component are three important parts of gene ontology (GO) (https://geneontology.org/docs/ontology-documentation/, accessed on 7 February 2024). GO and Kyoto Encyclopedia of Genes and Genomes (KEGG) (https://www.genome.jp/kegg/pathway.html, accessed on 7 February 2024) pathway analyses were performed to predict a possible molecular mechanism of the analyzed compounds against cancer. Enrichment analyses were performed using ShinyGO v0.741 with adjusted *p*-value < 0.05. The top 10 terms identified were visualized in a diagram.

## 5. Conclusions

In summary, the presented in silico evaluations comprehensively characterize the prospective anticancer utility of 7α-acetoxy-6β-hydroxyroyleanone and its derivatives. The compounds exhibit favorable pharmacological attributes alongside projected targeting of proliferation, survival, and stability pathways dysregulated in cancer. Strategic structural modifications appear to allow for the enhancement of anticancer properties. Specifically, benzoylated analogs showed enhanced potential compared to the lead compound Roy. Overall, the multifactorial computational data provide a robust foundation for the experimental validation of these compounds via in vitro studies, advancing their development as molecules for anticancer properties.

## Figures and Tables

**Table 1:** Predicted acute oral toxicity of the screened compounds obtained using ProTox-II server.

Compounds	Predicted LD_50_ (mg/kg)	Predicted Toxicity Class	Prediction Accuracy (%)
**1**	1000	4	69.26
**2**	75	3	67.38
**3**	100	3	67.38
**4**	100	3	67.38
**5**	75	3	68.07
**6**	100	3	67.38

**Table 2:** Predicted antineoplastic and anticarcinogenic activity of the examined compounds obtained by using the PASS server.

Compounds	Antineoplastic Activity		Anticarcinogenic Activity	
	Pa Value	Pi Value	Pa Value	Pi Value
**1**	0.879	0.005	0.419	0.028
**2**	0.819	0.010	0.332	0.047
**3**	0.834	0.008	0.398	0.031
**4**	0.822	0.009	0.312	0.054
**5**	0.858	0.006	0.378	0.035
**6**	0.851	0.007	0.347	0.042

**Table 3:** Frontier molecular orbitals, gap value, and descriptors for the optimized structures of the compounds in the gas phase, obtained using the DFT method.

Compound	E_HOMO_ (eV)	E_LUMO_ (eV)	ΔE (eV)	I (eV)	A (eV)	χ (eV)	μ (eV)	η (eV)	S (eV^−1^)	ω (eV)	ΔN_max_
**1**	−6.885	−3.433	3.45	6.89	3.43	5.16	−5.16	1.73	0.29	7.71	2.99
**2**	−6.971	−3.579	3.39	6.97	3.58	5.28	−5.28	1.70	0.29	8.20	3.11
**3**	−7.167	−3.462	3.71	7.17	3.46	5.31	−5.31	1.85	0.27	7.62	2.87
**4**	−6.365	−2.862	3.50	6.37	2.86	4.61	−4.61	1.75	0.29	6.08	2.63
**5**	−7.252	−3.537	3.72	7.25	3.54	5.39	−5.39	1.86	0.27	7.83	2.90
**6**	−7.197	−3.412	3.79	7.20	3.41	5.30	−5.30	1.89	0.26	7.43	2.80

ΔE—ELUMO–EHOMO; I—ionization energy; A—electron affinity; χ—electronegativity; μ—chemical potential; η—global chemical hardness; S—global chemical softness; ω—global electrophilicity index; and ΔN_max_—maximum additional electric charge.

## Data Availability

The data presented in this study are available in article and Supplementary Materials.

## Supplementary Materials

The following supporting information can be downloaded at: https://www.mdpi.com/article/10.3390/molecules29081807/s1, Table S1: Predicted physicochemical properties of the screened compounds, obtained using the SwissADME server; Table S2: Predicted lipophilicity of the screened compounds obtained using the SwissADME server; Table S3: Predicted water solubility of the screened compounds obtained using the SwissADME server; Table S4: Predicted pharmacokinetics parameters of the screened compounds obtained the using SwissADME server; Table S5: Predicted drug-likeness, medicinal chemistry, and lead-likeness pharmacokinetics parameters of the screened compounds obtained using the SwissADME server; Table S6: Predicted organ toxicity and toxicological endpoints of the screened compounds obtained using the ProTox-II server; Table S7: Toxicological pathways: nuclear receptor signaling pathways predicted for the screened compounds obtained using the ProTox-II server (% probability); Table S8: Toxicological pathways: stress response pathways predicted for the screened compounds obtained using the ProTox-II server (% probability); Table S9: Predicted physicochemical properties of the screened compounds, obtained using the SwissADME server; Table S10: Predicted acute toxicity of the screened compounds obtained using the StopTox server. Table S11. The molecular docking results of compounds **1**–**6** against target proteins
