# Supplementary material for: Anticancer Effects of Abietane Diterpene 7α-Acetoxy-6β-hydroxyroyleanone from Plectranthus grandidentatus and Its Semi-Synthetic Analogs: An In Silico Computational Approach
Source: Molecules. 2024 Apr 16;29(8):1807. doi: 10.3390/molecules29081807 (PMC11052076; doi:10.3390/molecules29081807)
Supplement: Supplementary file 1 [file molecules-29-01807-s001.zip › molecules-2924901-supplementary.pdf]

Supplementary materials

Table S1. Key physicochemical parameters of the examined compounds obtained using the SwissADME server.

**Compound**	Formula	MW	No. heavy atoms	No. aromatic heavy atoms	Fraction Csp3	No. ratable bonds	No. H-bond acceptors	No. H-bond donors	Molar refractivity
1	C22H30O6	390.47	28	0	0.68	3	6	2	104.48
2	C30H36O7	508.60	37	6	0.53	7	7	1	138.29
3	C30H36O7	508.60	37	6	0.53	6	7	1	138.67
4	C27H38O2	490.59	35	0	0.70	7	8	1	129.74
5	C26H36O7	460.56	33	0	0.69	7	7	1	123.42
6	C36H38O8	598.68	44	12	0.42	9	8	0	163.34

Table S2. Predicted lipophilicity of the examined compounds obtained using the SwissADME server.

**Compound**	TPSA	iLOGP	XLOGP3	WLOGP	MLOGP	Silicos-IT LogP	Consensus LogP
1	100.90	3.11	3.36	3.04	1.43	3.22	2.83
2	106.97	4.03	5.06	4.27	2.75	5.26	4.27
3	106.97	4.48	5.49	4.65	3.02	5.39	4.61
4	116.20	4.15	4.86	4.44	2.02	4.22	3.94
5	106.97	3.76	4.30	3.83	2.20	4.57	3.73
6	113.04	5.17	7.36	6.21	4.13	6.44	5.86

Table S3. Predicted water solubility of the examined compounds obtained using the SwissADME server.

**Compound**	ESOL log S	ESOL solubility [mg/mL]	ESOL solubility [mol/L]	ESOL class	Ali log S	Ali solubility [mg/mL]	Ali solubility [mol/L]	Ali class	Silicos-IT log S	Silicos-IT solubility [mg/mL]	Silicos-IT solubility [mol/L]	Silicos-IT class
1	-4.18	2.58e-02	6.61e-05	Moderately soluble	-5,16	2.72e-03	6.97e-06	Moderately soluble	-3.61	9.58e-02	2.45e-04	Soluble

2	-5.84	7.37e-04	1.45e-06	Moderately soluble	-7.05	4.55e-05	8.95e-08	Poorly soluble	-6.68	1.07e-04	2.10e-07	Poorly soluble
3	-6.18	3.39e-04	6.67e-07	Poorly soluble	-7.49	1.63e-05	3.20e-08	Poorly soluble	-6.66	1.11e-04	2.19e-07	Poorly soluble
4	-5.48	1.62e-03	3.30e-06	Moderately soluble	-7.03	4.53e-05	9.23e-08	Poorly soluble	-4.73	9.18e-03	1.87e-05	Moderately soluble
5	-4.94	5.26e-03	1.14e-05	Moderately soluble	-6.26	2.53e-04	5.50e-07	Poorly soluble	-5.01	4.54e-03	9.86e-06	Moderately soluble
6	-7.80	9.57e-06	1.60e-08	Poorly soluble	-9.56	1.64e-07	2.74e-10	Poorly soluble	-8.94	6.84e-07	1.14e-09	Poorly soluble

Table S4. Predicted pharmacokinetics parameters of the examined compounds obtained by using the SwissADME server.

**Compound**	GI adsorption	BBB permeant	Pgp substrate	CYP1A2 inhibitor	CYP2C19 inhibitor	CYP2C9 inhibitor	CYP2D6 inhibitor	CYP3A4 inhibitor	Log Kp [cm/s]
1	High	No	Yes	No	No	No	No	Yes	-6.30
2	High	No	Yes	No	No	Yes	No	Yes	-5.81
3	High	No	Yes	No	No	Yes	No	Yes	-5.50
4	High	No	Yes	No	No	Yes	No	No	-5.84
5	High	No	Yes	No	No	Yes	No	No	-6.06
6	Low	No	Yes	No	No	Yes	No	Yes	-4.73

Table S5. Predicted drug-likeness, medicinal chemistry, and lead-likeness pharmacokinetic parameters of the examined compounds obtained by using the SwissADME server.

**Compound**	Lipinski # violations	Ghose # violations	Veber # violations	Egan # violations	Muegge # violations	Bioavailability score	PAINS # alerts	Brenks # alerts	Leadlikeness # violations	Synthetic accessibility
1	0	0	0	0	0	0.56	1	1	1	5.23
2	1	3	0	0	1	0.56	1	2	2	5.92
3	1	3	0	0	1	0.56	1	2	2	5.82
4	0	2	0	0	0	0.56	1	2	2	5.99
5	0	0	0	0	0	0.56	1	2	2	5.89
6	1	4	0	1	1	0.56	1	2	3	6.19

Table S6. Predicted organ toxicity and toxicological endpoints activity of the examined compounds obtained using the ProTox-II server. (% probability)

Compound	Hepatotoxicity (% probability)	Carcinogenicity (% probability)	Immunotoxicity (% probability)	Mutagenicity (% probability)	Cytotoxicity (% probability)
1	Inactive (72)	Active (50)	Active (94)	Inactive (85)	Inactive (81)
2	Inactive (76)	Active (51)	Active (87)	Inactive (85)	Inactive (72)
3	Inactive (70)	Active (50)	Active (88)	Inactive (84)	Inactive (83)
4	Inactive (62)	Active (52)	Active (98)	Inactive (77)	Inactive (85)
5	Inactive (77)	Active (53)	Active (94)	Inactive (87)	Inactive (71)
6	Inactive (57)	Active (51)	Active (96)	Inactive (78)	Inactive (85)

Table S7. Toxicological pathways: nuclear receptor signaling pathways predicted for the examined compounds obtained using the ProTox-II server. (% probability)

Compound	Aryl Hydrocarbon Receptor (AhR)	Androgen Receptor (AR)	Androgen Receptor Ligand Binding Domain (AR-LBD)	Aromatase	Estrogen Receptor Alpha (ER)	Estrogen Receptor Ligand Binding Domain (ER-LBD)	Peroxisome Proliferator Activated Receptor Gamma (PPAR-Gamma)
1	Inactive (91)	Inactive (86)	Inactive (87)	Inactive (87)	Inactive (77)	Inactive (87)	Inactive (96)
2	Inactive (94)	Inactive (75)	Inactive (7)	Inactive (83)	Inactive (68)	Inactive (85)	Inactive (96)
3	Inactive (89)	Inactive (86)	Inactive (85)	Inactive (83)	Inactive (75)	Inactive (83)	Inactive (94)
4							
5	Inactive (97)	Inactive (74)	Inactive (80)	Inactive (82)	Inactive (73)	Inactive (89)	Inactive (97)
6	Inactive (86)	Inactive (86)	Inactive (82)	Inactive (82)	Inactive (72)	Inactive (78)	Inactive (95)

Table S8. Toxicological pathways: stress response pathways predicted for the compounds examined obtained using the ProTox-II server. (% probability)

Compound	Nuclear Factor (Erythroid-Derived 2-Like 2/Antioxidant Responsive Element) (nrf2/ARE)	Heat Shock Factor Response Element (HSE)	Mitochondrial Membrane Potential (MMP)	Phosphoprotein (Tumor Supressor) p53	ATPase Family AAA Domain Containing Protein 5 (ATAD5)

1	Inactive (76)	Inactive (76)	Inactive (58)	Inactive (93)	Inactive (84)
2	Inactive (84)	Inactive (84)	Inactive (56)	Inactive (90)	Inactive (90)
3	Inactive (84)	Inactive (84)	Inactive (52)	Inactive (89)	Inactive (85)
4	Inactive (74)	Inactive (74)	Active (52)	Inactive (84)	Inactive (86)
5	Inactive (76)	Inactive (76)	Active (50)	Inactive (95)	Inactive (93)
6	Inactive (74)	Inactive (74)	Active (53)	Inactive (86)	Inactive (85)

Table S9. Predicted acute toxicity of the examined compounds obtained using the StopTox server.

**Compound**	Inhalation Toxicity	Oral Toxicity	Dermal Toxicity	Eye Irritation and Corrosion	Skin Sensitization	Skin Irritation and Corrosion
1	No	No	No	No	No	No
2	No	No	No	No	No	No
3	No	No	No	No	No	No
4	No	No	No	No	No	No
5	No	No	No	No	No	No
6	No	No	No	No	Yes	No

Table S10. Predicted toxicity risks for the examined compounds obtained using the OSIRIS server.

**Compound**	Mutagenic Potential	Tumorigenic Potential	Irritant Potential	Reproductive Effectivity
1	Medium	Low	High	Low
2	Medium	Low	High	Low
3	Medium	Low	High	Low
4	Medium	Low	High	Low
5	Medium	Low	High	Low
6	Medium	Low	High	Low

Table S11. The molecular docking results of compounds **1-6** against target proteins.

Compound	Binding energy (kcal/mol)	Hydrogen bonds	Other bonds
			**BCL-2**
**1**	-9.50	ASP B: 70	GLU B: 111, PHE B: 112, GLY B: 104, ALA B: 108, ARG B: 105, LEU B: 96, PHE B: 63, TYR B: 67, MET B: 74, PHE B: 71
**2**	-10.46	LEU B: 160	VAL B: 107, PHE B: 63, GLY B: 104, GLU B: 58, PRO B: 163, GLY B: 162, TYR B: 161, SER A: 76, ARG A: 69, ALA A: 72, GLY B: 60, ALA B: 59, ASP B: 62

**3**	-10.22	LEU B: 160	PHE B: 63, TYR B: 67, ARG B: 66, ASP B: 62, GLU B: 58, PRO B: 163, TYR B: 161, GLY B: 162, SER A: 76, ARG A: 69, ALA A: 72, ALA B: 59, GLY B: 60, VAL B: 107
**4**	-10.28	TYR B: 161	ASN B:102, ARG A: 68, ARG A: 105, PHE B: 63, VAL B: 107, ARG B: 66, ALA B: 59, ASP B: 62, PRO B: 163, GLU B: 58, SER A: 76, ALA A: 72, GLYB: 104, LEU B: 160, TYR B: 67
**5**	-8.71	ASP B: 62	GLY B: 60, VAL B: 107, GLY B: 104, PHE B: 63, ARG B: 66, SER A: 76, LEU B: 160, PRO B: 163, TYR B: 161, GLU B: 58, ALA B: 59
**6**	-10.36	TYR B: 161	ALA A: 72, GLU B: 58, ALA B: 59, ASP B: 62, VAL B: 107, PHE B: 63, ARG B: 66, TYR B: 67, GLY B: 104, ARG B: 105, TRP B: 103, PRO B: 163, SER A: 76, SER A: 75
			**BCL-XL**
**1**	-10.42	-	PHE A: 105, TYR A: 101, ARG A: 100, ASP B: 133, ARG A: 103, ARG B: 132, GLU B: 129, ALA A: 104, PHE B: 131, LEU B: 130, PHE B: 105, ALA B: 141, ASN B: 136, GLY B: 138, PHE B: 97
**2**	-10.95	-	PHE B: 97, GLY B: 138, PHE B: 105, LEU B: 108, ASN B: 135, SER B: 106, LEU B: 130, GLU B: 129, ALA A: 104, ARG B: 139, ASP B: 133, TYR A: 101, PHE A: 97; PHE A: 105, TYR B: 101
**3**	-12.26	ARG A: 139, ASN A: 136	LEU A: 108, GLU A: 129, ALA A: 142, LEU A: 130, ARG A: 102, ARG A: 132, ALA B: 104, TYR B: 101, GLY A: 138, PHE A: 97, PHE A: 105, SER A: 106
**4**	-12.84	-	ARG B: 102, GLU B: 129, ALA A: 104, ASP B: 133, TYR A: 101, ASN B: 136, GLY B: 138, TYR B: 101, PHE B: 105, PHE B: 97, ALA B: 142, SER B: 106, LEU B: 108, LEU B: 130, ASP B: 107, PHE B: 146
**5**	-9.94	-	ASP A: 133, PHE B: 97, TYR B: 101, PHE B: 105, ASN A: 136, TYR A: 101, GLY A: 138, PHE A: 105, PHE A: 97, LEU A: 130, ALA A: 142, ARG A: 139, ALA B: 104, ARG B: 100
**6**	-11.25	-	GLU A: 96, ALA A: 93, TYR A: 195, VAL A: 141, PHE A: 191, GLY A: 138, PHE A: 97, TYR A: 101, ARG A: 139, ALA A: 142, ARG B: 139, ALA A: 104, PHE B: 105, PHE A: 105, ASN A: 136
			**Caspase 3**
**1**	-9.09	TYR A: 197, PRO B: 201	LYS A: 137, GLY B: 202, TYR B: 203, ALA B: 200, TYR A: 195, VAL A: 266, MET A: 268, GLY A: 125, THR A: 140, ARG A: 164, LEU A: 136, GLU A: 124, ASP A: 135
**2**	-9.63	ARG A: 164, TYR A: 197	TYR B: 197, TYR B: 195, VAL B: 266, ARG B: 164, CYS B: 264, VAL A: 266, CYS A: 264, PRO B: 201, TYR A: 195, THR A: 140, LEU A: 136, GLY A: 125, LYS A: 137, GLU A: 124, GLU A: 124, GLU B: 124, MET B: 268, PRO A: 201
**3**	-9.58	TYR A: 197	THR B: 140, LYS B: 137, TYR B: 197, LEU B: 136, PRO A: 201, ARG B: 164, CYS B: 264, VAL A: 266, GLU A: 124, VAL B: 266, ARG A: 164, CYS A: 264, GLY A: 125, LEU A: 136, TYR A: 195, THR A: 140, GLY B: 202, LYS A: 137, GLU B: 124, PRO B: 201, GLY B: 125
**4**	-11.86	ARG A:164, TYR B:197	THR A:140, GLY A: 125, GLU A: 124, GLU B: 124, LEU B: 136, LYS B: 137, ASP B: 135, GLY B: 125, TYR B: 195, THR B: 140, PRO A: 201, MET B: 268, ARG B: 164, CYS A: 264, VAL B: 266, CYS B: 264, VAL A: 266, PRO B: 201, TYR A: 195, TYR A: 197, LEU A: 136, LYS A: 137,
**5**	-8.39	ARG A: 164, CYS A: 264	LEU B: 136, THR B: 140, TYR B: 195, MET B: 268, TYR B: 197, PRO A: 201, VAL B: 266, VAL A: 266, PRO B: 201, ARG B: 164, TYR A: 197, GLU B: 124, LYS B: 137, GLU A: 124, GLY B: 125, ASP B: 135
**6**	-9.89	LYS B: 137	LEU A:136, GLY A: 125, CYS B: 264, TYR A: 197, PRO B: 201, VAL A: 266, ARG B: 164, ARG B: 164, VAL B: 266, ARG A: 164, CYS A: 264,

			GLU A: 124, GLU B: 124, TYR B: 195, PRO A: 201, LEU B: 136, GLY A: 202, GLU A: 167, GLY B: 125
			**Caspase 9**
**1**	-10.60	THRB: 181, PROB: 349	THRB: 179, ARGB: 178, HISB: 237, CYSB: 287, ARGB: 180, PHEB: 351, SERB: 361, PHEB: 348, GLYB: 350, GLYB: 360, GLYB: 182, ASPB: 186, SERB: 183, PROB: 357
**2**	-8.43	GLY B: 350	LYS B: 394, PHE B: 348, PRO B: 349, TRP B: 354, ARG B: 355, PHE B: 351, PRO B: 357, ARG B: 178, THR B: 181, THR B: 179, ARG B: 180, CYS B: 287, HIS B: 237
**3**	-9.35	GLY B: 350, LYS B: 394	GLY B: 395, ILE B: 396, TYR B: 397, PHE B: 348, LYS B: 398, PRO B: 349, GLY B: 288, CYS B: 287, TRP B: 354, PHE B: 351
**4**	-10.30	TYR B: 397	TRP B: 354, PRO B: 357, PHE B: 351, GLY B: 350, LYS B: 394, TRP B: 362, PHE B: 348, GLY B: 395, ILE B: 396, PRO B: 349, LYS B: 398
**5**	-8.19	-	LYS B: 394, GLY B: 395, ILE B: 396, TYR B: 397, PRO B: 349, PHE B: 348, TRP B: 354, LYS B: 398, GLY B: 350, PHE B: 351
**6**	-9.63	-	PHE B: 348, ARG B: 355, TRP B: 354, PHE B: 351, PRO B: 357, ARG B: 178, THR B: 179, THR B: 181, ARG B: 180, HIS B: 237, CYS B: 287, GLY B: 288, PRO B: 239, GLY B: 350
			**CDK2**
**1**	-11.86	-	GLY A: 11, GLN A: 131, GLU A: 12, ASN A: 132, LYS A: 33, ASP A: 145, ALA A: 144, VAL A: 18, PHE A: 80, ALA A: 31, VAL A: 64, LEU A: 134, GLU A: 81, ILE A: 10, LEU A: 83, PHE A: 82, HIS A: 84, GLN A: 85, ASP A: 86
**2**	-12.18	GLN A: 131	LYS A: 129, ASN A: 132, GLY A: 13, GLU A: 12, GLY A: 11, VAL A: 18, LYS A: 33, ALA A: 144, ASP A: 145, PHE A: 80, ALA A: 31, VAL A: 64, PHE A: 82, GLU A: 81, ILE A: 10, LEU A: 83, GLN A: 85, LEU A: 134, ASP A: 86
**3**	-12.56	-	VAL A: 64, PHE A: 80, ALA A: 144, LYS A: 33, ASP A: 145, LEU A: 83, ALA A: 31, PHE A: 82, LEU A: 134, ILE A: 10, GLY A: 11, GLN A: 85, ASP A: 86, ASN A: 132, LYS A: 129, GLN A: 131, GLY A: 13, GLU A: 12, GLU A: 81, VAL A: 18
**4**	-13.49	-	LYS A: 20, VAL A: 18 GLY A: 11, GLN A: 131, GLU A: 12, GLYA: 13, ASP A: 145, ASN A: 132, LYS A: 33, ALA A: 144, VAL A: 64, PHE A: 80, ALA A: 31, LEU A: 134, LEU A: 83, ILE A: 10, PHE A: 82, LYS A:89, GLN A:85, ASP A: 86, LEU A: 298, HIS A: 84
**5**	-11.49	ASP A: 145, LYS A: 33	HIS A: 84, ASP A: 86, ILE A: 10, GLY A: 11, VAL A: 18, GLU A: 12, GLY A: 13, GLY A: 16, THR A: 14, ASN A: 132, ALA A: 144, VAL A: 64, GLN A: 131, LEU A: 134, ALA A: 31, GLN A: 85, LEU A: 83, PHE A: 82
**6**	-11.57	-	VA LA: 18 ILE A: 10, LEU A: 83, PHE A: 80, ALA A: 31, VAL A: 64, GLU A: 81, LEU A: 134, PHE A: 82, HIS A: 84, GLN A: 85, LYS A: 89, LYS A: 88, GLN A: 131, GLY A: 11, GLY A: 13, GLU A: 12
			**CDK6**
**1**	-11.36	VAL A: 150	LEU A: 32, PRO A: 35, LEU A: 34, ARG A: 30, ARG A: 82, ALA A: 149, ALA A: 152, LEU A: 151, SER A: 78, ILE A: 198, ALA A: 191, LEU A: 193, PRO A: 195, LEU A: 192, SER A: 194
**2**	-13.27	ALAA: 152, VALA: 150	ASP A: 81, ARG A: 52, SER A: 78, VAL A: 77, LEU A: 151, PRO A: 74, LEU A: 185, ALA A: 149, PRO A: 195, ILE A: 198, GLU A: 31, LEU A: 34, ALA A: 191, LYS A: 36, THR A: 38, LEU A: 192, LEU A: 440
**3**	-13.83	ALAA: 152, VALA: 150	SER A: 39, LEU A: 40, THR A: 38, LEU A:192, LEU A: 193, PRO A: 74, VAL A: 77, LEU A: 151, LEU A: 185, SER A: 78, ILE A: 198, ALA A: 149, PRA A:195, LEU A: 34, SER A: 194, GLE A: 31, LYS A: 36

**4**	-14.28	ASP A: 81	GLU A: 148, HISB: 67, ALA A: 149, ARG A: 82, LEU A: 151, PRO A: 74, ALA A: 152, PRO A: 195, SER A: 78, LEU A: 193, ILE A: 198, LEU A: 185, ALA A: 191, LEU A: 40, VAL A: 77, ARG A: 52, LYS A: 36, VAL A: 150, GLU A: 31, LEU A: 34, ARG A: 30, LEU A: 33
**5**	-12.14	LEU A: 193	TRP A: 41, LEU A: 40, ASP A: 81, ARG A: 82, LEU A: 34, SER A: 78, ILE A: 198, VAL A: 77, LEU A: 185, PRO A: 74, ALA A: 152, PRO A: 195, LEU A: 151, VAL A: 150, SER A: 194, GLU A: 31, LEU A: 192, ALA A: 191, THR A: 38, LYS A: 36, PRO A: 35, CYS A: 85
**6**	-15.45	-	LEU A: 32, PRO A: 35, GLU A: 31, VAL A: 150, ARG A: 30, LEU A: 34, ALA A: 149, ARG A: 82, ASP A: 81, SER A: 78, ILE A: 198, ALA A: 152, LEU A: 151, PRO A: 74, LEU A: 185, VAL A: 77, ARG A: 52, ALA A: 191, LEU A: 40, LEU A: 192, SER A: 194, PRO A: 195, LYS A: 36
		**EGFR**	
**1**	-10.04	CYS A: 751	ARG A: 817, CYS A: 773, GLY A: 772, LEU A: 694, MET A: 769, VAL A: 702, GLN A: 767, LEU A: 820, ALA A: 719, THR A: 830, MET A: 742, GLU A: 738, LYS A: 721, ASP A: 831
**2**	-11.14	THR A: 766	PHE A: 699, THR A: 830, LYS A: 721, MET A: 742, GLU A: 738, ASP A: 831, VAL A: 702, GLN A: 767, LEU A: 820, ALA A: 719, LEU A: 768, MET A: 769, LEU A: 694, GLY A: 772, CYS A: 773, ASN A: 818, ARG A: 817
**3**	-10.38	MET A: 769	GLY A: 695, PHE A: 699, VAL A: 702, LEU A: 694, GLY A: 772, ALA A: 719, LYS A: 721, LEU A: 768, THR A: 766, LEU A: 820, MET A: 742, THR A: 830 ARG A: 817, ASP A: 831
**4**	-11.65	-	ARG A: 817, PRO A: 853, ASP A: 813, LEU A: 834, GLU A: 734, LYS A: 851, GLY A: 833, ASP A: 737, VAL A: 741, LYS A: 836, ALA A: 835, ILE A: 735, GLU A: 738, PHE A: 832, LEU A: 723, ASP A: 831, PHE A: 699, ALA A: 698
**5**	-9.94	MET A: 769	GLY A: 695, VAL A: 702, ASP A: 831, THR A: 830, LEU A: 694, GLU A: 738, PHE A: 832, MET A: 742, LYS A: 721, THR A: 766, ALA A: 719, GLN A: 767, LEU A: 820, MEU A: 768, PRO A: 770, GLY A: 772, CYS A: 773, PHE A: 771
**6**	-10.54		ASP A: 831, PHE A: 699, GLY A: 695, VAL A: 702, THR A: 830, GLU A: 738, THR A: 766, MET A: 742, ALA A: 719, LEU A: 694, GLY A: 772, LEU A: 820, ASN A: 818
		**VEGFR**	
**1**	-10.25	ARG A: 1049, ASN A: 921, LEU A: 838, CYS A: 917	GLY A: 839, VAL A: 846, ALA A: 864, GLU A: 915, PHE A: 916, LYS A: 918, PHE A: 919, GLY A: 920, LEU A: 1033, PHE A: 1045
**2**	-11.50	GLU A: 883, ASP A: 1044	LYS A: 866, ILE A: 886, LEU A: 887, ILE A: 890, VAL A: 896, VAL A: 897, LEU A: 1017, CYS A: 1022, ILE A: 1023, HIS A: 1024, ARG A: 1025, ILE A: 1042, CYS A: 1043, PHE A: 1045, GLY A: 1046
**3**	-11.77	GLU A: 883, ASP A: 1044	HIS A: 814, LYS A: 866, ILE A: 886, LEU A: 887, HIS A: 889, ILE A: 890, VAL A: 896, VAL A: 897, LEU A: 1017, LYS A: 1021, CYS A: 1022, ILE A: 1023, HIS A: 1024, ARG A: 1025, ILE A: 1042, CYS A: 1043, PHE A: 1045, GLY A: 1046
**4**	-12.39	ILE A; 1023	ILE A: 886, VAL A: 897, LEU A: 887, GLU A: 883, VAL A: 914, CYS A: 1043, LEU A: 1033, PHE A: 1045, ILE A: 890, ASP A: 1044, LEU A: 1017, VAL A:896, ILE A:1042, HIS A: 1024, CYS A: 1022, ARG A: 1025
**5**	-9.95	GLU A: 883	PHE A: 843, LYS A: 866, ALA A: 879, LEU A: 880, SER A: 882, ILE A: 886, LEU A: 887, ILE A: 890, VAL A: 896, VAL A: 897, LEU A: 1017, CYS

			A: 1022, ILE A: 1023, HIS A: 1024, ARG A: 1025, ILE A: 1042, CYS A: 1043, ASP A: 1044
**6**	-11.03	ASP A: 1050, ARG A: 1049, LEU A: 1047, GLY A: 1046, ASN A: 1031	ALA A: 842, PHE A: 843, ASP A: 1026, ARG A: 1030, ASP A: 1044, PHE A: 1045, ALA A: 1048, ILE A: 1051, ALA A: 1063, ARG A: 1064, PRO A: 1066
		**P53**	
**1**	-9.86	GLN A: 23	ARG A: 10, PHE A: 16, ASN A: 17, LYS A: 20, ILE A: 21, ILE A: 22, GLY A: 24, GLU A: 89, TYR A: 92, LEU A: 100, LEU A: 103, ARG A: 104, CYS A: 114, PRO A: 115
**2**	-11.32	-	ARG A: 10, HIS A: 11, PHE A: 16, ASN A: 17, LYS A: 20, ILE A: 21, ILE A: 22, GLN A: 23, GLY A: 24, ARG A: 61, GLU A: 89, TYR A: 92, LEU A: 100, LEU A: 103, ARG A: 104, CYS A: 114, PRO A: 115, HIS A: 198, GLY A: 199, ALA A: 200, ARG A: 203, VAL A: 229
**3**	-11.06	ARG A: 203, ASN A: 17	ARG A: 10, PHE A: 16, LYS A: 20, ILE A: 21, ILE A: 22, GLN A: 23, GLU A: 89, TYR A: 92, LEU A: 100, GLY A: 199, ALA A: 200, VAL A: 229, THR A: 230, PRO A: 231, ASN A: 232, GLN A: 260
**4**	-11.40	LEU A:100, THR A: 230	GLU A: 89, TYR A: 92, ARG A: 104, VAL A: 229, ARG A: 203, GLY A: 199, PRO A: 231, ASN A: 232, LYS A: 20, ARG A: 10, ASN A: 17, ILE A: 21, PHE A: 16, ILE A: 22, ARG A: 61, GLN A: 23
**5**	-9.95	ARG A:203, ILE A:21, ASN A:17	ARG A: 10, PHE A: 16, LYS A: 20, ILE A: 22, GLN A: 23, GLU A: 89, TYR A: 92, LEU A: 100, GLY A: 199, ALA A: 200, VAL A: 229, THR A: 230, PRO A: 231, ASN A: 232
**6**	-10.33	ARG A: 10	PHE A: 16, ASN A: 17, LYS A: 18, LYS A: 20, ILE A: 21, ILE A: 22, GLN A: 23, GLU A: 89, TYR A: 92, LEU A: 100, ARG A: 203, SER A: 228, VAL A: 229, THR A: 230, PRO A: 231, ASN A: 232
		**PARP-1**	
**1**	-11.15	GLY A: 863	GLY A: 888, TYR A: 889, HIS A: 862, SER A: 904, ALA A: 898, LYS A: 903, PHE A: 897, TYR A: 896, GLU A: 988, MET A: 980
**2**	-11.15	ARG A: 878, ALA A: 880	PRO A: 881, ILE A: 879, TYR A: 896, GLY A: 894, ILE A: 895, TYR A: 889, HIS A: 862, GLY A: 876, SER A: 864, LEU A: 877, ILE A: 872, GLN A: 875, GLY A: 871, ASN A: 868
**3**	-11.08	ASN: 868, ARG: 878	SERA: 864, TYR A: 907, GLY A: 863, HIS A: 862, TYR A: 896, TYR A: 889, GLY A: 894, ALA A: 880, LEU A: 877, ILE A: 895, GLY A: 876, ILE A: 872, ARG A: 865, HIS A: 909
**4**	-11.66	ARG A: 878	PRO A: 881, ILE: A:879, TYR A: 889, ALA A: 880, GLY A: 894, TYR A: 896, GLN A: 875, HIS A: 862, LEU A: 877, GLY A: 871, GLY A: 876, ILE A: 895, ILE A: 872, ASN A: 868
**5**	-10.80	ASN A: 868, ALA A: 880, ARG A: 878	HIS A: 909, ARG A: 865, SER A: 864, TYR A: 907, GLY A: 863, HIS A: 862, TYR A: 896, ILE A: 879, TYR A: 889, GLY A: 894, LEU A: 877, ILE A: 895, GLY A: 876, ILE A: 872
**6**	-12.60	-	LEU A: 877, ILE A: 872, SER A: 864, HIS A: 862, ASN A: 868, GLY A: 863, GLU A: 988, ALA A: 898, SER A: 904, PHE A: 897, LYS A: 903, TYR A: 907, TYR A: 896, GLY A: 888, TYR A: 889, GLY A: 876, ARG A: 878, ILE A: 895
